# Roles of *Suaeda vermiculata* Aqueous-Ethanolic Extract, Its Subsequent Fractions, and the Isolated Compounds in Hepatoprotection against Paracetamol-Induced Toxicity as Compared to Silymarin

**DOI:** 10.1155/2021/6174897

**Published:** 2021-09-17

**Authors:** Salman A. A. Mohammed, Hussein M. Ali, Hamdoon A. Mohammed, Mohsen S. Al-Omar, Suliman A. Almahmoud, Mahmoud Z. El-Readi, Ehab A. Ragab, Ghassan M. Sulaiman, Mohamed S. A. Aly, Riaz A. Khan

**Affiliations:** ^1^Department of Pharmacology and Toxicology, College of Pharmacy, Qassim University, Qassim 51452, Saudi Arabia; ^2^Department of Biochemistry, Faculty of Medicine, Al-Azhar University, Assiut 71524, Egypt; ^3^Department of Medicinal Chemistry and Pharmacognosy, College of Pharmacy, Qassim University, Qassim 51452, Saudi Arabia; ^4^Department of Pharmacognosy, Faculty of Pharmacy, Al-Azhar University, Cairo 11371, Egypt; ^5^Department of Medicinal Chemistry and Pharmacognosy, Faculty of Pharmacy, JUST, Irbid 22110, Jordan; ^6^Department of Clinical Biochemistry, Faculty of Medicine, Umm Al-Qura University, Makkah 21955, Saudi Arabia; ^7^Department of Biochemistry, Faculty of Pharmacy, Al-Azhar University, Assiut 71524, Egypt; ^8^Division of Biotechnology, Department of Applied Sciences, University of Technology, Baghdad 10066, Iraq; ^9^Hospital of the Police Academy, Nasr City, Cairo 11765, Egypt

## Abstract

*Suaeda vermiculata*, a halophyte consumed by livestock, is also used by Bedouins to manage liver disorders. The aqueous-ethanolic extract of *S. vermiculata*, its subsequent fractions, and pure compounds, *i.e*., pheophytin-A (1), isorhamnetin-3-*O*-rutinoside (2), and quercetin (3), were evaluated for their hepatoprotective efficacy. The male mice were daily fed with either silymarin, plant aq.-ethanolic extract, fractions, pure isolated compounds, or carboxyl methylcellulose (CMC) for 7 days (*n* = 6/group, *p.o*.). On the day 7^th^ of the administrations, all, except the intact animal groups, were induced with hepatotoxicity using paracetamol (PCM, 300 mg/kg). The anesthetized animals were euthanized after 24 h; blood and liver tissues were collected and analysed. The serum aspartate transaminase (AST) and alanine transaminase (ALT) levels decreased significantly for all the *S. vermiculata* aq.-ethanolic extract, fraction, and compound-treated groups when equated with the PCM group (*p* < 0.0001). The antioxidant, superoxide dismutase (SOD), increased significantly (*p* < 0.05) for the silymarin-, *n*-hexane-, and quercetin-fed groups. Similarly, the catalase (CAT) enzyme level significantly increased for all the groups, except for the compound 2-treated group as compared to the CMC group. Also, the glutathione reductase (GR) levels were significantly increased for the *n*-butanol treated group than for the PCM group. The oxidative stress biomarkers, lipid peroxide (LP) and nitric oxide (NO), the inflammatory markers, IL-6 and TNF-*α*, and the kidney's functional biomarker parameters remained unchanged and did not differ significantly for the treated groups in comparison to the PCM-induced toxicity bearing animals. All the treated groups demonstrated significant decreases in cholesterol levels as compared to the PCM group, indicating hepatoprotective and antioxidant effects. The quercetin-treated group demonstrated significant improvement in triglyceride level. The *S. vermiculata* aq.-ethanolic extract, fractions, and the isolated compounds demonstrated their hepatoprotective and antioxidant effects, confirming the claimed traditional use of the herb as a liver protectant.

## 1. Introduction

Liver disorders inflict people on a larger scale, and millions suffer worldwide. Various indigenous systems of medicine recommend a plethora of herbs and other botanical-based medicaments for treating various types of liver disorders. Several symptomatic and clinical indications are related to malfunctioning of the liver, which generally are grouped as nonalcoholic liver disorders. Primarily, oxidative stress, hepatic inflammation, and liver steatosis are considered prime causes [1]. Synthetic products, herbs, and herbal admixtures with strong antioxidant activity have been shown to reduce the sufferings and symptoms thereby exerting hepatoprotective activity [2]. However, clear-cut pieces of evidence for the hepatoprotection efficacy of the majority of the herbs are seldom and sparse.

Nonetheless, the liver protective-activity-established herbs and natural products including silymarin, glycyrrhizin, and other plant-based products have been effectively utilized as herbal concoctions and drinks [3]. The need for the bioactivity confirmation, dose, and administering frequency standardizations, together with investigations of any predictive and speculative side-effects owing to the herbs' quality, dose, administration frequency, and mechanism of action are imperative. Consequent to the exponential increments in the use of complementary and alternative medicines, especially the herbals, also among the patients with liver disorders [4], the bioactivity standardization and safety evaluation exercises of the frequently used formulations are needed to be accelerated and well-established. In this regard, animal model-based studies have shown anti-inflammatory and antioxidation-based positive effects on the liver [5] which contributes to the improvement in the liver's functioning. The investigations of liver biochemistry of the oxidative, antilipid-peroxidative, and inflammatory marker manifestations in the hepatic tissue have been widely used as a comparative standard for confirming proper/normal functioning of the liver [6]. The toxicity controls and prevention and the botanicals and other herbal-related liver toxicity generation products' investigational studies play an important part in finding safe uses of the plant-based products [7]. Objectively defined bioactivity testing endpoints defined by the standardized parametric levels of biomarkers and other biochemical, physiological, and histological observations for the standardized herbal extracts and isolated pure compounds are indispensable to verify the hepatoprotective actions of the traditional herbal medicament with ample confidence. Definitive histopathological evidence as liver's functional and biologic improvements is a step further towards activity levels and toxicological safety determinations of the products. Mechanistically, the stimulated uptake of glucose reduced serum triglycerides and hepatic cholesterol increased mitochondrial activity and ATP production, along with the reduced catabolic reactions, leading to cholesterol, bile acids, and plasma membrane's lipid level reductions, as well as the compromised immunomodulation, have been part of the primary indicators in hepatoprotective investigations milieu [8].

The halophytic herb, *Suaeda vermiculata* Forssk, a member of the plant family Amaranthaceae, grows in central Saudi Arabia and other Mediterranean regions. The plant belongs to the desert halophyte category and is used by nomads as a liver-protecting agent [9–11]. Antimicrobial, antioxidant, and cytotoxic activities of *S. vermiculata* extract, fractions, and their isolated compounds are reported. The major porphyrin-class product of the plant, pheophytin-A, has been isolated and evaluated for its antioxidant and cytotoxic effects [9, 12–14].

Recently, dose-dependent hepatoprotective action of *S*. *vermiculata* aqueous- (aq.-) ethanolic extract in the carbon tetrachloride- (CCl_4_-) induced hepatotoxicity using rat models was demonstrated by us [14]. For the current study, the prophylactic action of the *S*. *vermiculata* aq.-ethanolic extract, its fractions, and isolated compounds on the paracetamol- (PCM-) induced liver toxicity in mice is investigated. The major constituents of the *n*-butanol (*n*-BuOH), ethyl acetate (EtOAc), and chloroform (CHCl_3_) fractions were isolated-purified, characterized, and bioactivity evaluated. Paracetamol (PCM), also known as acetaminophen, a widely used nonprescription analgesic and antipyretic drug, that does not demonstrate liver toxicity at therapeutic doses but at elevated doses causes hepatic and renal toxicity in humans and experimental animals. PCM was used experimentally to induce liver toxicity in animal models during the current study [15]. The PCM toxicity is responsible for 50% of the acute liver failure cases in western countries [16]. A single (over) dose of PCM is known to rapidly induce hepatotoxicity [17–19] in mice, which is biomechanistically similar to the effects in humans, and is regarded clinically as a robust model [20]. Hence, mice were the preferred model compared to rats which were highly resistant to PCM-induced hepatotoxicity [18]. In this context, the current study sets out to confirm the traditionally claimed hepatoprotective activity of the plant-based tea, decoctions, and other crude formulations on the PCM-induced liver toxicity in the animal models. The current study also evaluated the safety of the plant materials' uses at usually the higher doses as practiced by the herbalists, including Bedouins and the locals. The plant, *S. vermiculata*, extract's effects on the kidneys, liver, blood sugar, and lipid levels in addition to the antioxidant and anti-inflammation actions in the PCM-induced toxicity-bearing animal models were investigated. During our previous study [14], only aq.-ethanolic extract's activity was evaluated in the CCl_4_-induced hepatotoxic conditions using rat models, while the current study investigates the PCM-induced hepatotoxicity protection by the aq.-ethanolic and its subsequent fractions, *i.e*., *n*-hexane, chloroform, ethyl acetate, and *n*-butanol, together with the compounds isolated from these fractions. The study demonstrated the hepatoprotective efficacy of these isolated compounds, *i.e*., pheophytin-A, quercetin, and isorhamnetin-3-*O*-rutinoside, from CHCl_3_, EtOAc, and *n*-BuOH fractions of the plant, respectively. In addition to the liver biomarkers, antioxidants, superoxide dismutase (SOD), catalase (CAT), and glutathione reductase (GR), as well as oxidative stress markers, *i.e*., lipid peroxide (LP) and nitric oxide (NO), and inflammatory biomarkers (*i.e*., IL6 and TNF-*α* levels), were also investigated in *in vivo* experimental conditions.

## 2. Materials and Methods

### 2.1. Chemicals and Reagents

All chemicals were of analytical grade. Methanol (HPLC grade) and formic acid were purchased from Sigma-Aldrich, USA. Pure paracetamol was obtained from Dr. Amin Dervish, Department of Pharmaceutics, College of Pharmacy, Qassim University, Kingdom of Saudi Arabia; locally available silymarin tablets (Micro Labs Limited, Mumbai, India) were used as obtained.

### 2.2. Plant Materials, Extraction, Fractionation, and Column Chromatographic Separations of the Major Constituents

The plants' whole herbs were collected in October 2019 from Buraydah, Qassim, KSA, and identified by Prof. Dr. Ahmed El-Oglah, Department of Biological Sciences, Yarmouk University, Irbid, Jordan. The plant material was compared to the authentic sample available in the herbarium of the College of Pharmacy, Qassim University, under the herbarium deposit # 78. The plant material (1.5 kg) was dried in shade and grinded to a coarse powder, which was exhaustively extracted three times with 70% aqueous-ethanol (3 L × 3) using the cold maceration technique under stirring for 24 h for each cycle. The hydroalcoholic extract was filtered and evaporated to dryness under reduced pressure at a temperature < 40°C, yielding 86.3 g of the dried extract. Approximately, 50 g of the dried extract was suspended in 1 L of distilled water and fractionated between *n*-hexane, CHCl_3_, EtOAc, and *n*-BuOH, in sequence, which resulted in 6.5 g, 4.5 g, 7 g, and 11 g of *n*-hexane, CHCl_3_, EtOAc, and *n*-BuOH fractions, respectively. About 1.0 g of the CHCl_3_ fraction was chromatographed over Sigel column chromatography (Sigel CC) eluted with *n*-hexane : ethyl acetate (100 : 0 to 70 : 30) to give five subfractions of A to E. Subfraction A (250 mg) was further purified on sephadex LH-20 using methanol as eluent to give compound 1 (120 mg). About 2.0 g of the *n*-BuOH fraction was subjected to Sigel CC using CHCl_3_ : MeOH (90 : 10 to 60 : 40) as eluent to give three major fractions A, B, and C. Fraction B (780 mg) was subjected to sephadex LH-20 gel filtration chromatography, followed by Sigel CC to give seven fractions (BA-BG). Fraction BD (300 mg) was subjected to RP-C_18_ CC (Starta® C_18_-E50g/150 ml, Giga, USA) eluted with 40-80% methanol, followed by preparative TLC (CHCl_3_ : MeOH : H_2_O (80 : 20 : 2)) to yield compound 2 (170 mg) as a yellow amorphous powder. A part of the EtOAc fraction (2.0 g) was subjected to several Sigel CC and sephadex LH-20 filtration to yield compound 3 (135 mg) (Scheme 1, Supplementary file). The isolated compounds were subjected to ^1^H and ^13^C-NMR and HR-MS spectroscopic analyses to confirm their identities (Spectral data available in the Supplementary file).

### 2.3. Acute Toxicity Studies and Sample Size

Acute toxicity was performed according to the OECD guidelines [21, 22]. In brief, 12-weeks-old male mice (*n* = 25), weighing 20 ± 5 g and overnight fasted, were randomly given single 4 g/kg dose of either aq.-ethanol extract, or *n*-hexane, CHCl_3_, EtOAc, and *n*-BuOH fractions (*n* = 5/group) through oral (*p.o*.) route. Mice were monitored for abnormal conduct and movements during the first three days while any deaths were followed up to 2 weeks [22]. The experiments' required sample size was established by the mean ± SEM and AST values of the PCM-induced hepatotoxic and PCM-induced hepatotoxicity-treated animal groups, as reported earlier [23]. A two-tail option provided effect size *d* as 4.27 on G Power V.3.1.9.4 software [24], while to obtain the statistical power (1-*β* err prob) of 80% and a specific *α* error probability of 0.05, the least animal size per group was *n* > 3.

### 2.4. Experimental Animal Groups

The study was conducted as per the Animal Research: Reporting *In vivo* Experiments (ARRIVE) statement [25].

#### 2.4.1. Hepatoprotective Effect of *S. vermiculata* Aqueous-Ethanolic Extract, Fractions, and Isolated Compounds in PCM-Induced Liver Toxicity

Male, 8-weeks-old, naïve C57BL/6 mice (*n* = 66), weighing 20 ± 5 g, were obtained from the animal house facility, College of Pharmacy, Qassim University, Saudi Arabia, with 3 mice/cage, one week before the beginning of the animal studies. The animals were maintained at 25°C with a relative humidity of ~65%. The institutional Research Ethics Committee approved the experimental procedure and the animal care (Approval ID 2019-CP-8), as per the Guidelines for the Care and Use of Laboratory Animals. Mice were distributed at random into nine groups (*n* = 6/group). The intact mice (group I) was not treated, while the other mouse groups received *p.o.* once daily with 0.5% carboxyl methylcellulose (CMC, negative control, group II), 100 mg/kg silymarin (positive control, group III), 100 mg/kg pheophytin-A (group IV), 100 mg/kg isorhamnetin-3-*O*-rutinoside (group V), 100 mg/kg quercetin (group VI), or 400 mg/kg aq.-ethanolic extract, 400 mg/kg *n*-butanol fraction, 400 mg/kg ethyl acetate fraction, 400 mg/kg chloroform fraction, and 400 mg/kg *n*-hexane fraction (groups VII-XI), for 7 days, followed by the induction of hepatotoxicity in the overnight fasted animals [26] using single intraperitoneal (*i.p.*) dose of the PCM (300 mg/kg) [17–19, 26] that was dissolved in warm normal saline (i). Twenty-four hours after PCM administration, blood [27] and tissue samples were collected from the sacrificed animals [17, 28].

Percentage hepatotoxic protection was determined using the formula below [29]:

(1) $$\begin{array}{c} \text{Hepatoprotection}\%=\left[\frac{a-b}{a-c}\right]\times 100, \end{array}$$

where *a*, *b*, and *c* are the mean ± SEM of hepatotoxin, toxin treated with the tested sample, and control, respectively.

The liver tissues were homogenized, and the supernatant was obtained for measuring oxidant, antioxidant, and inflammatory markers.

### 2.5. Estimation of Serum Levels of AST, ALT, TP, and Creatinine

The concentration of ALT, AST, TP, and creatinine (Crescent Diagnostics, KSA; #CZ902L for ALT, #CZ904L for AST, and # 604 for creatinine) in plasma samples was estimated as described earlier [14].

### 2.6. Determination of Serum Levels of Glucose, Cholesterol, and Triglycerides

Glucose, cholesterol, and triglyceride levels (Crescent Diagnostics Company; #605, #603, and #611) were measured according to the method described earlier [14].

### 2.7. Determination of Oxidants and Antioxidant Levels

The CAT enzymatic activity was assayed in serum by the colorimetric method wherein the catalase reacted with the known amount of excess hydrogen peroxide. The remaining hydrogen peroxide reacts with 3,5-dichloro-2-hydroxybenzene sulfonic acid and 4-aminophenazone forming a chromophore that gave color at 520 nm [30]. The SOD levels in the liver tissue were assayed also using a colorimetric method which depended upon the enzyme's potency in inhibiting the phenazine methosulfate-mediated reduction of the nitro blue tetrazolium dye. The absorbances were measured at 560 nm for 5 minutes for control and tissue samples [31]. The GR was assayed in liver-tissue samples by another colorimetric method based on reducing the dithio-bis-2-nitrobenzene acid with reduced glutathione, producing a yellow color that was checked at 405 nm [32]. Lipid peroxide (malondialdehyde) levels were determined in serum samples by the colorimetric method, where the reaction between the malondialdehyde and thiobarbituric acid under acidic conditions at 95°C for 30 minutes produced thiobarbituric acid-based pink products, which were assayed at 534 nm [33]. Serum NO was also measured by colorimetric determination that represented one of the final products of the NO *in vivo* conditions, in addition to the nitrate. The addition of Griess reagents converted nitrite into a deep purple azo compound that was measured at 540 nm [34]. All the oxidant and antioxidant reagents were provided by the Biodiagnostic Company, Cairo, Egypt.

### 2.8. Determination of Interleukin 6 (IL6) and Tumor Necrosis Factor-Alpha (TNF-*α*)

IL6 and TNF-*α* were assayed in liver tissue homogenates by ELISA (enzyme-linked immunosorbent assay) kits (Cloud Clone Corp Company, USA). The microplate's measurements were at 450 nm (Microplate Reader, BioTek Instruments, Inc., Winooski, VT, USA).

### 2.9. Statistical Analysis

Data are represented as the mean ± standard error of the mean (SEM). Two-way ANOVA followed by a post hoc Tukey multigroup comparison assessed variations among the groups, and *p* < 0.05 was considered significant on GraphPad Prism 8.0.2. [35]. Normality of the data was obtained using the Kolmogorov–Smirnov test.

## 3. Results and Discussion

### 3.1. Isolation and Structure Elucidation of Major Constituents

**Figure 1:**
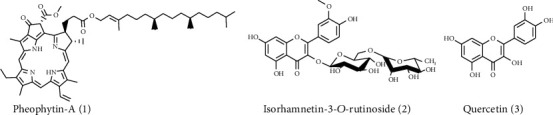
Chemical structures of the isolated compounds [1–3].

The whole plant, *S. vermiculata*, was used for aq.-ethanolic extraction, followed by further fractionations of the aq.-ethanolic extract into different solvent-based fractions. The major constituents were isolated by repetitive column chromatographic (CC) purification techniques involving silica gel-based normal, and reverse-phase (RP) silica gel-based CCs, preparative TLC (Thin Layer Chromatography), and finally gel filtration (Sephadex LH-20) techniques. The compounds, pheophytin-A (1), isorhamnetin-3-*O*-rutinoside (2), and quercetin (3), isolated from various fractions, were fully characterized by their ^1^H and ^13^C NMR spectral data and the HR-MS analyses and their comparison with the reported values. The isolation and characterization of compound 1, pheophytin-A, followed the previously reported method [9]. Compound 2 was isolated in a pure form as yellow amorphous powder from the *n*-BuOH fraction. The ^1^H NMR spectrum of compound 2 showed the proton signal pattern for the C3-glycosylated flavonols by exhibiting two single protons at *δ*_H_ 6.21 and 6.41 assigned for the C6 and C8 protons of the flavonol structure, respectively. The ABX system of protons resonating at *δ*_H_ 7.95 (br s), *δ*_H_ 7.64 (d, *J* =8.5 Hz), and *δ*_H_ 6.94 (dd, *J* = 2.0 and 8.5 Hz) showed their presence at C2′, C5′, and C6′, respectively. The ^1^H NMR spectrum also exhibited two proton doublets at *δ*_H_ 5.22 (*J* = 7.2 Hz) and *δ*_H_ 4.54 (*J* = 1.5 Hz), assigned to the glucose and rhamnose anomeric protons, respectively. The ^13^C NMR spectrum of compound 2 resembled the carbon signal pattern of the glycosylated flavonol based upon comparison with literature data. In addition, HR-MS analyses of compound 2 showed a molecular ion peak (M^+.^) as [M-H]^−^ at *m*/*z* 623.16113 (C_28_H_32_O_16_); therefore, the compound was identified as a flavonol glycoside; isorhamnetin-3-*O*-rutinoside (2) [36, 37]. Compound (3) was isolated from the EtOAc fraction as a yellowish powder. The ^1^H and ^13^C NMR spectral data were typically identical to the reported values for quercetin [38]. HR-MS analysis confirmed the compound's identity, which showed the molecular ion peak at *m*/*z* 303.04924 [M+H]^+^, compatible with the molecular formula of quercetin. The presence of compounds 1-3 (Figure 1) has been confirmed by the previous LC-MS analysis of the *S. vermiculata* aq.-ethanolic extract. The relatively higher occurrences of these compounds (1, 2, and 3) as confirmed by the LC-MS in the aq.-ethanolic extract were at 23.69, 4.37, and 12.45%, respectively [14].

### 3.2. Acute Toxicity and Dose Selection

Among all the animal groups, 2 EtOAc and 1 *n*-hexane fraction-fed mice died on days 2 and 3 of the dose administrations, respectively, during oral drug administrations. The acute toxicity results conducted for 2 weeks were similar to the previously reported [14]. The results indicated safety at the administered dose. Corresponding to Hedge and Sterner scale, 10% (400 mg/kg) of the given dose was chosen for further experiments [39].

### 3.3. Hepatoprotective Activity of the S. vermiculata Aqueous-Ethanolic Extract, Fractions, and Isolated Compounds

For the hepatoprotective experiments, the AST and ALT biomarkers, the negative control group was significantly elevated (*p* < 0.0001) compared to the intact group (Table 1). The increased ALT and AST enzymatic levels were attributed to hepatic cell damage and necrosis due to the liver toxicity induced by the PCM, as also reported in previous studies. Also, the PCM toxicity led to reactive oxygen species (ROS) and LP releases causing oxidative stress [40, 41]. All the *S. vermiculata* aq.-ethanolic extract and fraction-fed groups showed a significant decrease in both the ALT and AST enzymatic activities as compared with the PCM alone group. Similarly,the isolated compounds; pheophytin-A, isorhamnetin-3-*O*-rutinoside, and quercetin fed-groups demonstrated significant reductions in ALT and AST levels than the PCM-fed group demonstrated significant reductions in ALT and AST levels than the PCM-fed group. The administrations of these materials demonstrated hepatoprotective effects as indicated by the improvements in liver functions, and a significant decrease (*p* < 0.05) in liver-enzyme activities, as compared to the PCM-fed injury group, was observed. The protective effects of the aq.-ethanolic extract and fractions may be attributed to their antioxidative as well as anti-inflammatory effects. The renal biomarkers, *i.e*., urea, and creatinine levels remained unchanged. The TP (total proteins), creatinine, and glucose level values also remained nearly unchanged in *S. vermiculata* aq.-ethanolic extract and fraction-fed groups or the isolated compounds, *i.e*., pheophytin-A (1), isorhamnetin-3-*O*-rutinoside (2), and quercetin (3), in comparison to the negative group, as competitively maintained near the referral standard product, silymarin, a well-known liver-protecting natural product (Tables 1 and 2).

The PCM-fed group demonstrated significantly increased cholesterol and decreased triglyceride levels as compared with the intact group. The PCM toxicity caused oxidative stress and lipid peroxidation, leading to increased cholesterol levels. These results are in conformity with the previously reported study [42]. All the treated groups demonstrated decreased cholesterol levels than the PCM-induced toxic animal group, thereby indicating the hepatoprotective and antioxidative effects of the plant materials. The quercetin-treated group demonstrated significant improvement in triglyceride level compared to the PCM-fed group. The silymarin, aq.-ethanolic extract, and the isorhamnetin-3-*O*-rutinoside groups also demonstrated improvements in triglyceride levels, but these were not significant. The improvements in lipid profile in the quercetin-treated group were attributed to its hepatoprotective and antioxidant effects, as also mentioned by a similar study reported earlier [43].

**Figure 2:**
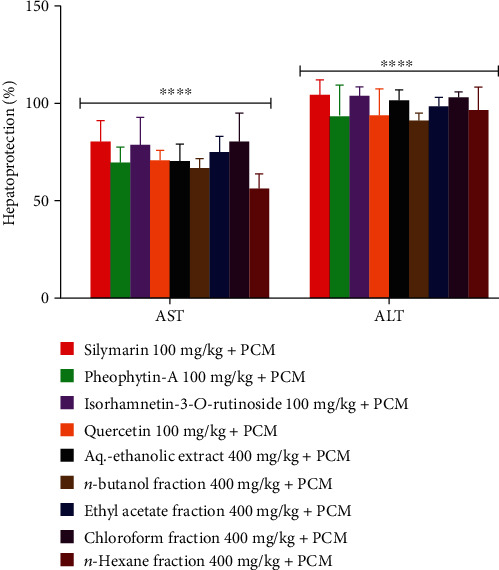
Percentage protection after PCM-induced elevations of AST and ALT enzyme levels in the negative and intact groups at 0 and 100% protection, respectively, and thus are not included in the above graph. Values denoted are the mean ± SEM and ^∗∗∗∗^*P* < 0.001 for the groups when equated to the negative group.

The total hepatoprotective percentage for the liver markers was also determined. The PCM-induced negative control animals' group (CMC (carboxyl methylcellulose)) was considered at 0% protection, while the intact group was considered to have 100% hepatoprotection. The hepatoprotection percentage was observed by measuring various biomarkers according to their maintained levels and compared with the controls (Figure 2). There is maintenance of the percent levels of the biomarkers in the aq.-ethanolic extract, fractions, and the isolated product-fed groups, as compared to the referral standard, silymarin. It confirmed the hepatoprotective properties of the aq.-ethanolic extract, fractions, and isolated products of the plant, *S. vermiculata*, which is in full consonant with the traditionally claimed hepatoprotective effects of the plant, including from the previous study that demonstrated the hepatoprotection using *S. vermiculata* ethanolic extract in CCl_4_-induced liver injury models [14].

### 3.4. Antioxidant Activity of S. vermiculata Aqueous-Ethanolic Extract, Fractions, and Isolated Compounds

Oxidative stress is considered to play significant roles in PCM-induced liver and renal damages in experimental animals [44, 45]. The oxidative stress is mitigated using endogenous antioxidants, or free radical scavengers, *e.g*., plant extracts, and flavonoids [46]. As compared to the intact group, the PCM-administered group demonstrated significantly lowered antioxidant SOD and CAT enzymatic activity levels, which are crucial in ROS elimination. The significant decreases in the SOD and CAT activities demonstrated depleted antioxidant potentials in the PCM group which was attributed to the consumption of SOD and CAT during ROS detoxification [47]. The isolated compounds, quercetin (3) of *S. vermiculata*, silymarin, and *n*-hexane fraction, demonstrated significantly higher SOD values than the PCM group. Similarly, isolated compounds (1 and 3), aq.-ethanolic extract, all fractions, and silymarin-fed groups demonstrated significantly increased CAT enzymatic activity as compared to the PCM group. The quercetin's increased SOD and CAT enzymatic activity levels were in agreement with the previous study confirming its antioxidant role [47]. According to the previous study, the PCM-administered group demonstrated significantly reduced GR levels, as compared to the intact group [48]. The aq.-ethanolic extract and *n*-butanol fraction-treated groups demonstrated a significant increase in the GR levels, as compared to the PCM-fed group, while the remaining groups, including silymarin, did not improve the GR levels, as compared to the PCM-fed group. The NO is reported to demonstrate a peak value 24 hr after the PCM administration [45], and the current NO data is in conformity with the previous data demonstrating a significant increase in the PCM group when equated to the intact group. All the tested and treated groups did not demonstrate any significant difference in NO as compared to PCM. The LP which triggers cellular injury through membrane enzyme and receptor deactivation, including protein cross-linking and fragmentation [49], was statistically not different among all the studied groups (Table 3). The previous study reported by us also showed the antioxidant effects of the *S. vermiculata* ethanolic extract through *in vitro* free radical scavenging parameter [14], while the current result demonstrated the *in vivo* antioxidant effects of the plant aq.-ethanolic extract, fractions, and the isolated compounds. The antioxidant effects of *S. vermiculata* were also previously reported [50], and the extracts' antioxidant effect was attributed to the high contents of flavonoids and related polyphenols [51]. Similarly, the antioxidant activity of quercetin was also detected in both the *in vivo* and *in vitro* PCM-induced liver toxicity models [43]. Nonetheless, the elevated NO levels lead to deleterious reaction with superoxide anion (O_2_^−^) thereby generating peroxynitrite radical (ONOO−). The simultaneous production of NO and O_2_^−^ is expected during the inflammation and other pathological conditions, while ONOO^−^, being a potent oxidant, attacks multiple biological targets [52]. The NO scavenging effects on O_2_^−^ are suggested to be a mechanism by which the host tissues are protected from the deleterious effects of O_2_^−^ and O_2_^−^-derived ROS [53].

### 3.5. Effect of S. vermiculata on Inflammatory Markers

The inflammatory cytokines, IL6 and TNF-*α*, respectively, are vital to the *β*-cell functional regulation. Their increased levels are associated with the elicitation of various diseases [54]. For the PCM-induced liver toxicity model, the levels of these cytokines are reported to increase, indicating the advancement of liver damages [55]. During the current study, the TNF-*α* values decreased significantly in the PCM-fed group than in the intact group, while all the treated groups, including silymarin, demonstrated no change in its levels when compared to the PCM-fed group. The IL-6 did not demonstrate any significant difference among all the groups as compared to the PCM-fed group (Table 4). As observed earlier, the administration of ethanolic extract of *S. vermiculata* in a carrageenan-induced paw edema inflammation model decreased the inflammation [14, 56]. In contrast, in the current study, the inflammatory marker IL6 and TNF-*α* levels remained insignificant for the aq.-ethanolic extract as well as other fractions in addition to the 3 isolated compounds from the *S. vermiculata*.

Previous studies have demonstrated that one-week preadministration of the extract before the PCM-induced liver toxicity does not interfere with the P450 activity required for acetaminophen metabolism [57]. In our previous study, the effect of aq.-ethanolic extract after one-week administration on normal animals did not demonstrate any significant changes in the liver, kidney, and cardiac markers as compared to the intact animals [14]. There are no studies available that demonstrate hepatoprotection from PCM-induced liver toxicity using pheophytin-A and the flavonol glycoside, isorhamnetin-3-*O*-rutinoside in mice. This is the first report in this connection. However, quercetin has previously been demonstrated to improve liver markers, antioxidant activities, and reduction of LP and inflammatory markers in liver toxicity [47, 58, 59]. The current study also reiterates the quercetin role in liver protection.

## 4. Conclusion

The paracetamol (PCM) overdose induced liver injury and caused hepatic cell damage through elevations of AST and ALT enzymatic activities as compared to the intact group observed. The PCM group also increased the oxidative stress by elevating the nitric oxide (nitrite) levels and decreasing the antioxidant SOD, CAT, and GR levels as compared with the control. The TNF-*α* values also decreased significantly in the PCM-induced toxicity group when compared to the intact group. Thus, the current study demonstrated the hepatoprotective potency of the *S. vermiculata* aq.-ethanolic extract, fractions, and their major constituents, *i.e*., pheophytin-A, a flavonol glycoside, isorhamnetin-3-*O*-rutinoside, and quercetin. The aq.-ethanolic extract, fraction, and the isolated compound, protective effects were confirmed by significant reductions in AST and ALT enzymatic activities as equated to the PCM-fed toxicity-bearing mice. The aq.-ethanolic extract, fraction, and the isolated compound treatments also decreased the oxidative stress induced by the PCM-generated toxicity through the elevation of antioxidant enzymatic activities of SOD and CAT. Also, the improvement of lipid profile with no adverse effects on the liver, kidney, and glucose markers was observed. The ongoing data on the potentials of the antioxidants, levels of biomarkers, and comparable liver-protective effects, equated to the referral standard, silymarin, confirmed the plant's role in hepatoprotection. The safety of the extract and fractions, at higher doses, also confirmed the plant materials to be safe. Therefore, the plant materials' consumption by locals can be considered nontoxic within the tested dose level.

## Figures and Tables

**Table 1:** Effects of *S. vermiculata* aq.-ethanolic extract, fractions, and isolated compounds on liver functions of the PCM-induced liver toxicity in the experimental mice^∗^.

Animal groups	AST IU/L	ALT IU/L	TP g/dL
I. Intact control (no CMC, no extract/no fractions, no PCM)	64.11 ± 2.59^C^	55.53 ± 11.82^B^	5.07 ± 0.68^A^
II. Negative control (vehicle CMC 0.5%)+PCM	293.05 ± 52.28^A^	407.05 ± 105.06^A^	4.96 ± 0.19^A^
III. Silymarin 100 mg/kg+PCM	108.26 ± 10.40^B,C^	39.14 ± 7.44^B^	5.08 ± 0.40^A^
IV. Pheophytin-A 100 mg/kg+PCM	133.28 ± 7.66^B,C^	77.44 ± 15.73^B^	4.99 ± 0.26^A^
V. Isorhamnetin-3-*O*-rutinoside 100 mg/kg+PCM	112.54 ± 14.51^BC^	40.75 ± 4.49^B^	5.20 ± 0.35^A^
VI. Quercetin 100 mg/kg+PCM	129.44 ± 4.15^B,C^	76.09 ± 13.41^B^	5.70 ± 0.21^A^
VII. Aqueous-ethanolic extract 400 mg/kg+PCM	131.19 ± 8.90^B,C^	49.61 ± 5.64^B^	6.76 ± 1.18^A^
VIII. *n*-Butanol fraction 400 mg/kg+PCM	138.27 ± 4.58^C^	84.88 ± 3.59^B^	5.00 ± 0.76^A^
IX. Ethyl acetate fraction 400 mg/kg+PCM	119.63 ± 7.35^B,C^	60.10 ± 4.15^B^	5.72 ± 0.60^A^
X. Chloroform fraction 400 mg/kg+PCM	107.50 ± 13.56^B,C^	44.35 ± 2.71^B^	4.93 ± 0.30^A^
X1. *n*-Hexane fraction 400 mg/kg+PCM	164.52 ± 7.95^B^	66.29 ± 10.96^B^	4.30 ± 0.32^A^

^∗^Values denoted are the mean ± SEM. AST: aspartate transaminase; ALT: alanine transaminase; TP: total protein; CMC: carboxyl methylcellulose; PCM: paracetamol. Mean ± SEM not sharing the letters (A–C) in the respective column (AST, ALT, and TP) are significantly different (*p* < 0.0001). Raw data is available in the Supplementary file (Table S1).

**Table 2:** Effects of *S. vermiculata* aq.-ethanolic extract, fractions, and isolated compounds on kidney functions, blood glucose, triglycerides, and cholesterol of the PCM-induced liver toxicity in mice^∗^.

Animal groups	Creatinine mg/dL	Urea mg/dL	Glucose mg/dL	Cholesterol mg/dL	Triglycerides mg/dL
I. Intact control (no CMC, no extract/no fractions, no PCM)	0.62 ± 0.03^B^	48.83 ± 2.47^BC^	61.53 ± 5.25^B^	106.30 ± 7.65^B^	101.63 ± 19.62^A^
II. Negative control (vehicle CMC 0.5%)+PCM	0.67 ± 0.03^A,B^	55.01 ± 2.31^A,B,C^	67.26 ± 4.55^A,B^	148.44 ± 13.23^A^	55.14 ± 6.41^B,C,D^
III. Silymarin 100 mg/kg+PCM	0.86 ± 0.07^A,B^	85.39 ± 17.38^A,B^	44.27 ± 3.56^A,B^	93.50 ± 3.97^B,C^	79.98 ± 18.75^A,B,C,D^
IV. Pheophytin-A 100 mg/kg+PCM	0.78 ± 0.02^A,B^	92.73 ± 19.39^A^	50.24 ± 5.29^B^	90.80 ± 6.31^B,C^	54.52 ± 3.64^B,C,D^
V. Isorhamnetin-3-*O*-rutinoside 100 mg/kg+PCM	0.86 ± 0.03^A,B^	52.46 ± 1.56^A,B,C^	62.56 ± 7.10^A,B^	88.35 ± 3.30^B,C^	94.01 ± 11.75^A,B,C^
VI. Quercetin 100 mg/kg+PCM	0.84 ± 0.06^A,B^	51.57 ± 1.60^A,B,C^	67.26 ± 4.55^A,B^	148.90 ± 8.97^A^	105.55 ± 5.54^B^
VII. Aqueous-ethanolic extract 400 mg/kg+PCM	0.67 ± 0.19^A,B^	59.44 ± 4.50^A,B,C^	64.15 ± 4.59^B^	68.01 ± 5.74^C,D^	91.69 ± 12.23^A,B,C^
VIII. *n*-Butanol fraction 400 mg/kg+PCM	0.90 ± 0.07^A^	47.87 ± 1.95^B,C^	99.14 ± 13.23^A^	50.03 ± 2.01^D^	50.09 ± 1.21^C,D^
IX. Ethyl acetate fraction 400 mg/kg+PCM	1.03 ± 0.23^A^	40.15 ± 2.33^C^	63.74 ± 10.56^A,B^	99.15 ± 6.39^B,C^	58.16 ± 3.58^A,B,C,D^
X. Chloroform fraction 400 mg/kg+PCM	0.94 ± 0.06^A^	50.08 ± 4.50^B,C^	54.27 ± 3.88^B^	96.82 ± 11.47^B,C^	40.07 ± 2.08^D^
X1. n-Hexane fraction 400 mg/kg+PCM	0.92 ± 0.04^A^	72.74 ± 9.08^A,B,𝐶^	43.25 ± 7.53^A,B^	87.02 ± 5.89^B,C^	40.76 ± 1.10^D^

^∗^Values denoted are the mean ± SEM. Mean ± SEM not sharing the letters (A–D) in the respective column are significantly different (*p* < 0.05). Raw data is available in the Supplementary file (Table S2).

**Table 3:** Effects of *S. vermiculata* aq.-ethanolic extract, fractions, and isolated compounds on antioxidant activity in PCM-induced liver toxicity in experimental mice^∗^.

Groups	CAT U/g	SOD U/g	GR mg/g	LP nmol/g	NO *μ*mol/g
I. Intact control (no CMC, no extract/no fractions, no PCM)	993.17 ± 21.42^A,B^	210.60 ± 5.07^A^	0.37 ± 0.02^A^	6.85 ± 0.56^A^	0.45 ± 0.05^C^
II. Negative control (vehicle CMC 0.5%)+PCM	638.59 ± 13.89^C^	118.75 ± 4.18^C,D,E^	0.12 ± 0.02^C,D^	5.82 ± 1.25^A^	0.95 ± 0.05^A,B^
III. Silymarin 100 mg/kg+PCM	862.72 ± 12.51^A,B^	190.40 ± 13.39^A,B^	0.07 ± 0.02^D,E^	5.94 ± 0.35^A^	N.D.
IV. Pheophytin-A 100 mg/kg+PCM	829.29 ± 26.27^B^	134.84 ± 2.28^C,D^	0.03 ± 0.01^E^	8.06 ± 0.21^A^	1.31 ± 0.05^A^
V. Isorhamnetin-3-*O*-rutinoside 100 mg/kg+PCM	449.34 ± 18.93^D^	143.92 ± 10.04^C^	0.04 ± 0.00^E^	5.51 ± 0.95^A^	1.30 ± 0.15^A^
VI. Quercetin 100 mg/kg+PCM	1020.50 ± 17.92^A,B^	212.52 ± 3.12^A^	0.03 ± 0.00^E^	7.33 ± 0.31^A^	N.D.
VII. Aqueous-ethanolic extract 400 mg/kg+PCM	895.42 ± 77.53^A,B^	96.57 ± 10.32^D,E^	0.05 ± 0.00^D,E^	7.97 ± 0.21^A^	0.94 ± 0.05^B^
VIII. *n*-Butanol fraction 400 mg/kg+PCM	946.61 ± 33.64^A,B^	156.72 ± 9.13^B,C^	0.26 ± 0.03^B^	8.35 ± 0.16^A^	N.D.
IX. Ethyl acetate fraction 400 mg/kg+PCM	949.17 ± 7.05^A,B^	115.31 ± 9.76^C,D,E^	0.16 ± 0.01^C^	8.31 ± 1.02^A^	N.D.
X. Chloroform fraction 400 mg/kg+PCM	961.10 ± 28.83^A,B^	87.67 ± 3.58^E^	0.05 ± 0.01^D,E^	7.09 ± 0.73^A^	N.D.
X1. *n*-Hexane fraction 400 mg/kg+PCM	848.19 ± 72.40^A,B^	206.39 ± 16.84^A^	0.06 ± 0.01^D,E^	7.08 ± 0.46^A^	N.D.

^∗^Values denoted are the mean ± SEM. CMC: carboxyl methylcellulose; PCM: paracetamol; CAT: catalase; LP: lipid peroxide; SOD: superoxide dismutase; NO: nitric oxide; GR: glutathione reductase; N.D.: not determined. Mean ± SEM not sharing the letters (A–E) in the respective column (CAT, LP, SOD, NO, and GR) are significantly different (*p* < 0.05). Raw data is available in the Supplementary file (Table S3).

**Table 4:** Effects of *S. vermiculata* aq.-ethanolic extract, fractions, and isolated compounds on inflammatory markers in PCM-induced liver toxicity in experimental mice^∗^.

Groups	IL-6 pg/g	TNF-*α* pg/g
I. Intact control (no CMC, no extract/no fractions, no PCM)	6117.63 ± 33.57^A^	7570.44 ± 34.82^A^
II. Negative control (vehicle CMC 0.5%)+PCM	5919.70 ± 86.77^A,B^	7108.14 ± 32.36^B^
III. Silymarin 100 mg/kg+PCM	6031.83 ± 33.25^A,B^	7306.10 ± 77.72^A,B^
IV. Pheophytin-A 100 mg/kg+PCM	5852.54 ± 71.40^B^	7152.60 ± 21.56^B^
V. Isorhamnetin-3-*O*-rutinoside 100 mg/kg+PCM	5995.02 ± 80.45^A,B^	7304.19 ± 43.91^A,B^
VI. Quercetin 100 mg/kg+PCM	5956.74 ± 54.92^A,B^	7366.28 ± 96.33^A,B^
VII. Aqueous-ethanolic extract 400 mg/kg+PCM	6167.27 ± 23.56^A^	7329.63 ± 86.92^A,B^
VIII. *n*-Butanol fraction 400 mg/kg+PCM	5877.38 ± 18.22^B^	7138.10 ± 45.15^B^
IX. Ethyl acetate fraction 400 mg/kg+PCM	5903.53 ± 33.53^A,B^	7071.76 ± 24.95^B^
X. Chloroform fraction 400 mg/kg+PCM	6070.63 ± 87.33^A,B^	7344.04 ± 101.51^A,B^
X1. *n*-Hexane fraction 400 mg/kg+PCM	6015.82 ± 28.20^A,B^	7324.78 ± 59.08^A,B^

^∗^Values denoted are the mean ± SEM. CMC: carboxyl methylcellulose; PCM: paracetamol; IL6: interleukin-6; TNF-*α*: tumor necrosis factor-alpha. Mean ± SEM not sharing the letters (A–B) in the respective column (IL-6, TNF-*α*) are significantly different (*p* < 0.05). Raw data is available in the Supplementary file (Table S4).

## Data Availability

All the data were provided in the manuscript and supplementary file.

## Supplementary Materials

Supplementary Materials Supplementary file includes four tables (Tables S1 to S4) that describe the raw data related to the demonstrated biological activities of S. vermiculata. The file also includes nine figures (Figures S1 to S9) that showed the NMR and mass spectra of the isolated compounds. Besides, one scheme that describes the extraction and chromatographic separation of isolated compounds is also provided in the Supplementary file.
