# Supplementary material for: Roles of Suaeda vermiculata Aqueous-Ethanolic Extract, Its Subsequent Fractions, and the Isolated Compounds in Hepatoprotection against Paracetamol-Induced Toxicity as Compared to Silymarin
Source: Oxid Med Cell Longev. 2021 Sep 17;2021:6174897. doi: 10.1155/2021/6174897 (PMC8463249; doi:10.1155/2021/6174897)
Supplement: Supplementary Materials — Supplementary file includes four tables (Tables S1 to S4) that describe the raw data related to the demonstrated biological activities of S. vermiculata. The file also includes nine figures (Figures S1 to S9) that showed the NMR and mass spectra of the isolated compounds. Besides, one scheme that describes the extraction and chromatographic separation of isolated compounds is also provided in the Supplementary file. [file 6174897.f1.zip › Figure S2.pdf]

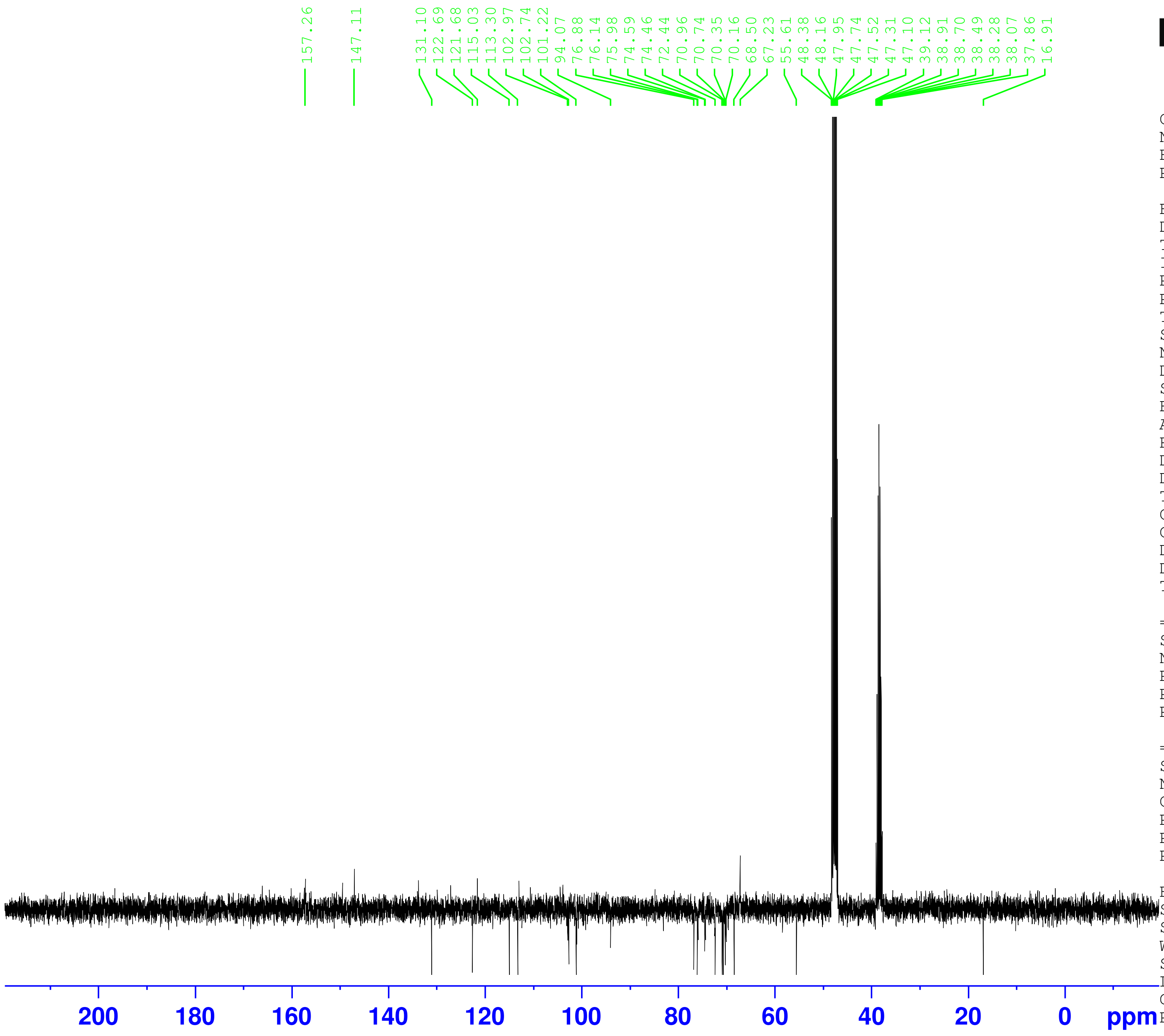

Current Data Parameters

NAME	msh-HC
EXPNO	2
PROCNO	1

F2 - Acquisition Parameters

Date_	20200901
Time	8.41
INSTRUM	spect
PROBHD	5 mm PABBO BB/
PULPROG	jmod
TD	65536
SOLVENT	MeOD
NS	14329
DS	4
SWH	24038.461 Hz
FIDRES	0.366798 Hz
AQ	1.3631488 sec
RG	205.37
DW	20.800 usec
DE	6.50 usec
TE	300.0 K
CNST2	145.0000000
CNST11	1.0000000
D1	2.00000000 sec
D20	0.00689655 sec
TD0	1

======== CHANNEL f1 ========

SFO1	100.6278593 MHz
NUC1	13C
P1	10.00 usec
P2	20.00 usec
PLW1	47.00000000 W

======== CHANNEL f2 ========

SFO2	400.1516006 MHz
NUC2	1H
CPDPRG[2	waltz16
PCPD2	90.00 usec
PLW2	18.00000000 W
PLW12	0.34722000 W

F2 - Processing parameters

SI	32768
SF	100.6177975 MHz
WDW	EM
SSB	0
LB	1.00 Hz
GB	0
PC	1.40
