# Supplementary material for: Roles of Suaeda vermiculata Aqueous-Ethanolic Extract, Its Subsequent Fractions, and the Isolated Compounds in Hepatoprotection against Paracetamol-Induced Toxicity as Compared to Silymarin
Source: Oxid Med Cell Longev. 2021 Sep 17;2021:6174897. doi: 10.1155/2021/6174897 (PMC8463249; doi:10.1155/2021/6174897)
Supplement: Supplementary Materials — Supplementary file includes four tables (Tables S1 to S4) that describe the raw data related to the demonstrated biological activities of S. vermiculata. The file also includes nine figures (Figures S1 to S9) that showed the NMR and mass spectra of the isolated compounds. Besides, one scheme that describes the extraction and chromatographic separation of isolated compounds is also provided in the Supplementary file. [file 6174897.f1.zip › Figure S8.pdf]

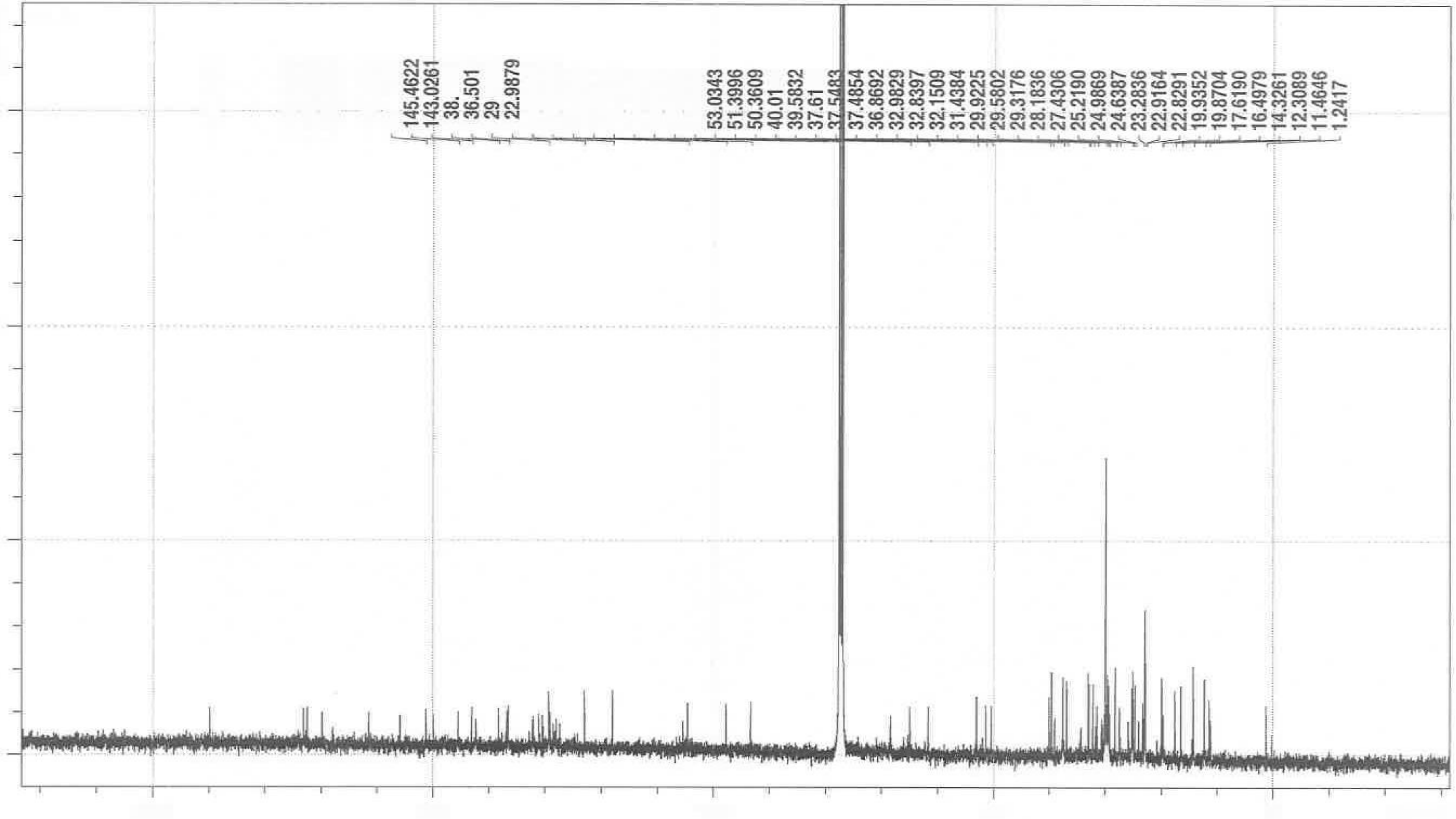
145.4622
143.0261
38.
36.501
29
22.9879
53.0343
51.3996
50.3609
40.01
39.5832
37.61
37.5483
37.4854
36.8692
32.9829
32.8397
32.1509
31.4384
29.9225
29.5802
29.3176
28.1836
27.4306
25.2190
24.9869
24.6387
23.2836
22.9164
22.8291
19.9352
19.8704
17.6190
16.4979
14.3261
12.3089
11.4646
1.2417
