# Supplementary material for: Roles of Suaeda vermiculata Aqueous-Ethanolic Extract, Its Subsequent Fractions, and the Isolated Compounds in Hepatoprotection against Paracetamol-Induced Toxicity as Compared to Silymarin
Source: Oxid Med Cell Longev. 2021 Sep 17;2021:6174897. doi: 10.1155/2021/6174897 (PMC8463249; doi:10.1155/2021/6174897)
Supplement: Supplementary Materials — Supplementary file includes four tables (Tables S1 to S4) that describe the raw data related to the demonstrated biological activities of S. vermiculata. The file also includes nine figures (Figures S1 to S9) that showed the NMR and mass spectra of the isolated compounds. Besides, one scheme that describes the extraction and chromatographic separation of isolated compounds is also provided in the Supplementary file. [file 6174897.f1.zip › Revised Supplementary File (1).docx]

Table S1: Raw data of Table 1: Effects of *S. vermiculata* extracts and isolated compounds on liver functions on the PCM-induced liver toxicity in experimental mice

|  |  |  |  |  |  |  |  |  |  |  |  |
| --- | --- | --- | --- | --- | --- | --- | --- | --- | --- | --- | --- |
| **AST** | Intact control | Negative control | Silymarin 100 mg/kg + PCM | Pheophytin-A 100 mg/kg + PCM | Flavonol glycoside 100 mg/kg + PCM | Quercetin 100 mg/kg + PCM | Mother Liquor 400 mg/kg + PCM | n-Butanol Extract 400 mg/kg + PCM | Ethyl Acetate Extract 400 mg/kg + PCM | Chloroform Extract 400 mg/kg + PCM | n-Hexane Extract 400 mg/kg + PCM |
|  | 55.216 | 204.736 | 76.16 | 102 | 156.128 | 133.28 | 121.856 | 125.664 | 110.432 | 156.128 | 175.168 |
|  | 68.544 | 191.224 | 102.816 | 135 | 156.128 | 143 | 118.048 | 133.6 | 144.704 | 118.048 | 186.592 |
|  | 68.544 | 221.321 | 114.24 | 156 | 72.352 | 114.24 | 167.552 | 141.04 | 133.28 | 53.312 | 159.936 |
|  | 59.445 | 434 | 152.32 | 124 | 89.31 | 136.24 | 146.7 | 128.19 | 95.2 | 106.624 | 134.74 |
|  | 61.447 | 479 | 94 | 146.21 | 94.37 | 125.24 | 108.69 | 154.87 | 124.47 | 98.67 | 151.47 |
|  | 71.448 | 228 | 110 | 136.48 | 106.94 | 124.65 | 124.27 | 146.28 | 109.69 | 112.21 | 179.24 |
|  | **64.11** | **293.05** | **108.26** | **133.28** | **112.54** | **129.44** | **131.19** | **138.27** | **119.63** | **107.50** | **164.52** |
|  | **6.34** | **128.05** | **25.47** | **18.77** | **35.54** | **10.16** | **21.80** | **11.22** | **17.99** | **33.21** | **19.47** |
|  | **6.00** | **6.00** | **6.00** | **6.00** | **6.00** | **6.00** | **6.00** | **6.00** | **6.00** | **6.00** | **6.00** |
|  | **2.59** | **52.28** | **10.40** | **7.66** | **14.51** | **4.15** | **8.90** | **4.58** | **7.35** | **13.56** | **7.95** |
|  |  |  |  |  |  |  |  |  |  |  |  |
| **% hepatoprotection** | | 0 | 80.71601 | 69.78488 | 78.84565 | 71.46218 | 70.70027 | 67.60425 | 75.74818 | 81.04681 | 56.13819 |
|  |  |  |  |  |  |  |  |  |  |  |  |
| **ALT** | Intact control | Negative control | Silymarin 100 mg/kg + PCM | Pheophytin-A 100 mg/kg + PCM | Flavonol glycoside 100 mg/kg + PCM | Quercetin 100 mg/kg + PCM | Mother Liquor 400 mg/kg + PCM | n-Butanol Extract 400 mg/kg + PCM | Ethyl Acetate Extract 400 mg/kg + PCM | Chloroform Extract 400 mg/kg + PCM | n-Hexane Extract 400 mg/kg + PCM |
|  | 41.888 | 674.016 | 68.544 | 19.04 | 34.272 | 34.272 | 30.464 | 83.776 | 57.12 | 45.696 | 68.544 |
|  | 41.888 | 129.472 | 22.848 | 69.056 | 53.312 | 91.392 | 68.544 | 96.24 | 76.16 | 53.312 | 15.232 |
|  | 15.232 | 245 | 26.656 | 134.24 | 26.656 | 41.888 | 49.504 | 79.994 | 64.736 | 34.272 | 89.21 |
|  | 91.392 | 776.832 | 54.67 | 88.21 | 46.37 | 118.048 | 44.88 | 86.34 | 45.696 | 48.61 | 77.21 |
|  | 57.12 | 295.31 | 29.67 | 59.71 | 51.24 | 97.77 | 61.94 | 91.67 | 55.67 | 39.97 | 62.87 |
|  | 85.68 | 321.64 | 32.45 | 94.37 | 32.67 | 73.14 | 42.31 | 71.28 | 61.19 | 44.21 | 84.67 |
|  | **55.53** | **407.05** | **39.14** | **77.44** | **40.75** | **76.09** | **49.61** | **84.88** | **60.10** | **44.35** | **66.29** |
|  | **28.96** | **257.33** | **18.23** | **38.53** | **11.00** | **32.84** | **13.80** | **8.80** | **10.16** | **6.65** | **26.86** |
|  | **6.00** | **6.00** | **6.00** | **6.00** | **6.00** | **6.00** | **6.00** | **6.00** | **6.00** | **6.00** | **6.00** |
|  | **11.82** | **105.06** | **7.44** | **15.73** | **4.49** | **13.41** | **5.64** | **3.59** | **4.15** | **2.71** | **10.96** |
|  |  |  |  |  |  |  |  |  |  |  |  |
| **% hepatoprotection** |  | 0 | 104.6638 | 93.76853 | 104.2047 | 94.15335 | 101.686 | 91.65035 | 98.70218 | 103.1829 | 96.94007 |
|  |  |  |  |  |  |  |  |  |  |  |  |
| **TP** | Intact control | Negative control | Silymarin 100 mg/kg + PCM | Pheophytin-A 100 mg/kg + PCM | Flavonol glycoside 100 mg/kg + PCM | Quercetin 100 mg/kg + PCM | Mother Liquor 400 mg/kg + PCM | n-Butanol Extract 400 mg/kg + PCM | Ethyl Acetate Extract 400 mg/kg + PCM | Chloroform Extract 400 mg/kg + PCM | n-Hexane Extract 400 mg/kg + PCM |
|  | 4.72 | 5.39 | 6.62 | 4.22 | 4.99 | 6.1 | 10.65 | 4.99 | 5.96 | 5.24 | 4.86 |
|  | 3.14 | 5.05 | 5.38 | 5.38 | 4.83 | 5.69 | 4.61 | 6.1 | 4.58 | 5.02 | 3.5 |
|  | 5.52 | 5.21 | 4.33 | 5.49 | 4.96 | 5.74 | 5.85 | 4.88 | 4.77 | 5.21 | 3.7 |
|  | 6.84 | 4.58 | 4.33 | 4.17 | 6.29 | 6.4 | 3.21 | 3.2 | 7.98 | 4.06 | 5.21 |
|  | 3.26 | 5.3 | 5.67 | 5.64 | 6.12 | 5.24 | 9.64 | 2.91 | 6.84 | 5.94 | 3.56 |
|  | 6.94 | 4.23 | 4.13 | 5.01 | 4.01 | 5.04 | 6.61 | 7.94 | 4.21 | 4.12 | 4.94 |
|  | **5.07** | **4.96** | **5.08** | **4.99** | **5.20** | **5.70** | **6.76** | **5.00** | **5.72** | **4.93** | **4.30** |
|  | **1.67** | **0.46** | **0.98** | **0.65** | **0.86** | **0.51** | **2.88** | **1.87** | **1.48** | **0.72** | **0.79** |
|  | **6.00** | **6.00** | **6.00** | **6.00** | **6.00** | **6.00** | **6.00** | **6.00** | **6.00** | **6.00** | **6.00** |
|  | **0.68** | **0.19** | **0.40** | **0.26** | **0.35** | **0.21** | **1.18** | **0.76** | **0.60** | **0.30** | **0.32** |

Table S2: Raw data of Table 2: Effects of *S. vermiculata* extracts and isolated compounds on kidney functions, blood glucose, triglycerides, and cholesterol on the PCM-induced liver toxicity in mice

| **Crea** | Intact control | Negative control | Silymarin 100 mg/kg + PCM | Pheophytin-A 100 mg/kg + PCM | Flavonol glycoside 100 mg/kg + PCM | Quercetin 100 mg/kg + PCM | Mother Liquor 400 mg/kg + PCM | n-Butanol Extract 400 mg/kg + PCM | Ethyl Acetate Extract 400 mg/kg + PCM | Chloroform Extract 400 mg/kg + PCM | n-Hexane Extract 400 mg/kg + PCM |
| --- | --- | --- | --- | --- | --- | --- | --- | --- | --- | --- | --- |
|  | 0.55 | 0.91 | 0.81 | 0.71 | 0.82 | 0.79 | 0.51 | 0.961 | 0.79 | 0.86 | 0.81 |
|  | 0.66 | 0.94 | 0.62 | 0.69 | 0.9 | 0.8 | 0.75 | 0.71 | 0.9 | 0.93 | 0.89 |
|  | 0.61 | 0.61 | 0.81 | 0.76 | 0.8 | 0.93 | 0.91 | 0.76 | 1.2 | 0.9 | 0.95 |
|  | 0.57 | 0.6 | 0.8 | 0.76 | 0.93 | 0.76 | 0.84 | 1.04 | 0.71 | 0.78 | 0.85 |
|  | 0.59 | 0.66 | 0.76 | 0.8 | 0.85 | 0.85 | 1 | 1.04 | 0.66 | 0.85 | 0.9 |
|  | 0.71 | 0.71 | 1 | 0.76 | 0.88 | 0.95 | 0.66 | 0.9 | 1.47 | 1.04 | 1 |
|  |  |  |  |  |  |  |  |  |  |  |  |
|  | **0.62** | **0.74** | **0.80** | **0.75** | **0.86** | **0.85** | **0.78** | **0.90** | **0.96** | **0.89** | **0.90** |
|  | **0.06** | **0.15** | **0.12** | **0.04** | **0.05** | **0.08** | **0.18** | **0.14** | **0.32** | **0.09** | **0.07** |
|  | **6.00** | **6.00** | **6.00** | **6.00** | **6.00** | **6.00** | **6.00** | **6.00** | **6.00** | **6.00** | **6.00** |
|  | **0.02** | **0.06** | **0.05** | **0.02** | **0.02** | **0.03** | **0.07** | **0.06** | **0.13** | **0.04** | **0.03** |
|  |  |  |  |  |  |  |  |  |  |  |  |
|  |  |  |  |  |  |  |  |  |  |  |  |
| **Urea** | Intact control | Negative control | Silymarin 100 mg/kg + PCM | Pheophytin-A 100 mg/kg + PCM | Flavonol glycoside 100 mg/kg + PCM | Quercetin 100 mg/kg + PCM | Mother Liquor 400 mg/kg + PCM | n-Butanol Extract 400 mg/kg + PCM | Ethyl Acetate Extract 400 mg/kg + PCM | Chloroform Extract 400 mg/kg + PCM | n-Hexane Extract 400 mg/kg + PCM |
|  | 51.24 | 54.15 | 62.1 | 86.57 | 54.94 | 55.14 | 51.11 | 45.24 | 31.11 | 43.55 | 68.98 |
|  | 46.17 | 61.84 | 136.44 | 148.71 | 51.9 | 47.58 | 68.78 | 44.26 | 41.69 | 62.14 | 59.44 |
|  | 38.38 | 60.52 | 73.6 | 51.87 | 54.91 | 48.12 | 55.61 | 52.56 | 47.73 | 40.25 | 79.14 |
|  | 52.04 | 47.58 | 51.61 | 153.53 | 56.87 | 49.16 | 58.94 | 49.02 | 43.56 | 57.36 | 60.66 |
|  | 55.98 | 49.88 | 47.01 | 70.3 | 49.16 | 57.07 | 75.76 | 54.01 | 37.14 | 37.09 | 114.43 |
|  | 49.17 | 56.06 | 141.6 | 45.43 | 47 | 52.33 | 46.43 | 42.14 | 39.68 | 60.09 | 53.76 |
|  | **48.83** | **55.01** | **85.39** | **92.74** | **52.46** | **51.57** | **59.44** | **47.87** | **40.15** | **50.08** | **72.74** |
|  | **6.06** | **5.66** | **42.57** | **47.49** | **3.81** | **3.93** | **11.02** | **4.77** | **5.70** | **11.02** | **22.25** |
|  | **6.00** | **6.00** | **6.00** | **6.00** | **6.00** | **6.00** | **6.00** | **6.00** | **6.00** | **6.00** | **6.00** |
|  | **2.47** | **2.31** | **17.38** | **19.39** | **1.56** | **1.60** | **4.50** | **1.95** | **2.33** | **4.50** | **9.09** |
|  |  |  |  |  |  |  |  |  |  |  |  |
|  |  |  |  |  |  |  |  |  |  |  |  |
| **Glucose** | Intact control | Negative control | Silymarin 100 mg/kg + PCM | Pheophytin-A 100 mg/kg + PCM | Flavonol glycoside 100 mg/kg + PCM | Quercetin 100 mg/kg + PCM | Mother Liquor 400 mg/kg + PCM | n-Butanol Extract 400 mg/kg + PCM | Ethyl Acetate Extract 400 mg/kg + PCM | Chloroform Extract 400 mg/kg + PCM | n-Hexane Extract 400 mg/kg + PCM |
|  | 67.77 | 74.12 | 55.98 | 55.14 | 55.78 | 74.12 | 68.12 | 94.11 | 51.14 | 49.65 | 71.41 |
|  | 46.12 | 55.21 | 63.68 | 36.14 | 43.16 | 55.21 | 59.14 | 127.4 | 49.3 | 58.79 | 75.98 |
|  | 67.02 | 79.12 | 36.84 | 49.27 | 98.78 | 79.12 | 46.87 | 88.25 | 53.33 | 29.14 | 69.78 |
|  | 44.21 | 72.98 | 55.89 | 43.33 | 47.89 | 72.98 | 32.28 | 60.76 | 37.19 | 46.14 | 50.18 |
|  | 72.63 | 70.53 | 86.14 | 49.47 | 107.89 | 70.53 | 54.91 | 147.45 | 88.12 | 13.86 | 50 |
|  | 71.45 | 51.58 | 76.84 | 32.28 | 44.74 | 51.58 | 41.05 | 76.84 | 103.33 | 61.93 | 67.54 |
|  | **61.53** | **67.26** | **62.56** | **44.27** | **66.37** | **67.26** | **50.40** | **99.14** | **63.74** | **43.25** | **64.15** |
|  | **12.87** | **11.16** | **17.38** | **8.73** | **29.10** | **11.16** | **12.95** | **32.39** | **25.85** | **18.45** | **11.24** |
|  | **6.00** | **6.00** | **6.00** | **6.00** | **6.00** | **6.00** | **6.00** | **6.00** | **6.00** | **6.00** | **6.00** |
|  | **5.25** | **4.55** | **7.10** | **3.56** | **11.88** | **4.55** | **5.29** | **13.22** | **10.56** | **7.53** | **4.59** |
|  |  |  |  |  |  |  |  |  |  |  |  |
| **Chol** | Intact control | Negative control | Silymarin 100 mg/kg + PCM | Pheophytin-A 100 mg/kg + PCM | Flavonol glycoside 100 mg/kg + PCM | Quercetin 100 mg/kg + PCM | Mother Liquor 400 mg/kg + PCM | n-Butanol Extract 400 mg/kg + PCM | Ethyl Acetate Extract 400 mg/kg + PCM | Chloroform Extract 400 mg/kg + PCM | n-Hexane Extract 400 mg/kg + PCM |
|  | 99.67 | 177.64 | 84.37 | 88.19 | 84.61 | 137.64 | 76.84 | 51.11 | 112.98 | 121.45 | 91.23 |
|  | 91.7 | 128.52 | 89.65 | 94.77 | 98.19 | 139.35 | 71.36 | 53.98 | 84.48 | 94.14 | 84.51 |
|  | 84.48 | 155.98 | 94.58 | 79.55 | 76.53 | 146.94 | 64.88 | 41.25 | 92.42 | 139.35 | 79.94 |
|  | 105.98 | 184.12 | 83.75 | 69.31 | 92.37 | 184.84 | 55.6 | 55.14 | 84.48 | 79.42 | 63.54 |
|  | 134.3 | 96.75 | 99.64 | 113.36 | 83.75 | 122.02 | 50.54 | 49.82 | 97.78 | 80.87 | 101.81 |
|  | 121.68 | 147.65 | 109.03 | 99.64 | 94.67 | 162.37 | 88.81 | 48.88 | 122.74 | 65.7 | 101.08 |
|  | **106.30** | **148.44** | **93.50** | **90.80** | **88.35** | **148.86** | **68.01** | **50.03** | **99.15** | **96.82** | **87.02** |
|  | **18.73** | **32.41** | **9.73** | **15.48** | **8.10** | **21.99** | **14.08** | **4.93** | **15.65** | **28.10** | **14.44** |
|  | **6.00** | **6.00** | **6.00** | **6.00** | **6.00** | **6.00** | **6.00** | **6.00** | **6.00** | **6.00** | **6.00** |
|  | **7.65** | **13.23** | **3.97** | **6.32** | **3.31** | **8.98** | **5.75** | **2.01** | **6.39** | **11.47** | **5.89** |
|  |  |  |  |  |  |  |  |  |  |  |  |
| **Triglyceride** | Intact control | Negative control | Silymarin 100 mg/kg + PCM | Pheophytin-A 100 mg/kg + PCM | Flavonol glycoside 100 mg/kg + PCM | Quercetin 100 mg/kg + PCM | Mother Liquor 400 mg/kg + PCM | n-Butanol Extract 400 mg/kg + PCM | Ethyl Acetate Extract 400 mg/kg + PCM | Chloroform Extract 400 mg/kg + PCM | n-Hexane Extract 400 mg/kg + PCM |
|  | 97.34 | 55.19 | 89.14 | 62.98 | 79.66 | 96.47 | 74.61 | 49.64 | 63.11 | 37.64 | 43.18 |
|  | 13.04 | 34.78 | 79.45 | 48.11 | 59.13 | 93.04 | 94.67 | 49.98 | 64.35 | 43.67 | 38.41 |
|  | 95.65 | 50.43 | 53.04 | 58.61 | 135.65 | 109.87 | 118.24 | 54.97 | 40.87 | 46.96 | 41.24 |
|  | 150.43 | 68.7 | 164.35 | 40.87 | 88.14 | 114.78 | 47.83 | 51.67 | 62.61 | 33.91 | 37.39 |
|  | 128.7 | 77.39 | 32.17 | 53.04 | 80 | 93.04 | 84.35 | 46.09 | 59.78 | 42.61 | 40 |
|  | 124.62 | 44.35 | 61.74 | 63.48 | 121.45 | 126.09 | 130.43 | 48.78 | 58.26 | 35.65 | 44.35 |
|  | **101.63** | **55.14** | **79.98** | **54.52** | **94.01** | **105.55** | **91.69** | **50.19** | **58.16** | **40.07** | **40.76** |
|  | **48.05** | **15.70** | **45.93** | **8.92** | **28.78** | **13.57** | **29.95** | **2.97** | **8.77** | **5.10** | **2.70** |
|  | **6.00** | **6.00** | **6.00** | **6.00** | **6.00** | **6.00** | **6.00** | **6.00** | **6.00** | **6.00** | **6.00** |
|  | **19.62** | **6.41** | **18.75** | **3.64** | **11.75** | **5.54** | **12.23** | **1.21** | **3.58** | **2.08** | **1.10** |

Table S3: Raw data of Table 3: Effects of *S. vermiculata* extracts and isolated compounds on anti-oxidant activity in PCM-induced liver toxicity in experimental mice

| **CAT** | Intact control | Negative control | Silymarin 100 mg/kg + PCM | Pheophytin-A 100 mg/kg + PCM | Flavonol glycoside 100 mg/kg + PCM | Quercetin 100 mg/kg + PCM | Mother Liquor 400 mg/kg + PCM | n-Butanol Extract 400 mg/kg + PCM | Ethyl Acetate Extract 400 mg/kg + PCM | Chloroform Extract 400 mg/kg + PCM | n-Hexane Extract 400 mg/kg + PCM |
| --- | --- | --- | --- | --- | --- | --- | --- | --- | --- | --- | --- |
|  | 1024.00 | 650.00 | 809.29 | 803.18 | 385.09 | 984.00 | 986.55 | 811.74 | 929.10 | 886.31 | 498.78 |
|  | 924.00 | 662.00 | 862.00 | 781.17 | 456.00 | 967.00 | 524.45 | 942.00 | 944.99 | 957.00 | 855.75 |
|  | 1067.00 | 591.00 | 894.00 | 794.00 | 512.00 | 1067.00 | 1054.00 | 1028.00 | 936.00 | 1057.00 | 942.00 |
|  | 946.00 | 602.00 | 851.00 | 814.37 | 426.31 | 996.00 | 896.00 | 1018.00 | 946.94 | 896.27 | 926.37 |
|  | 987.00 | 651.24 | 872.00 | 826.37 | 489.67 | 1041.00 | 924.24 | 984.24 | 976.24 | 1028.32 | 879.37 |
|  | 1011.00 | 675.28 | 888.00 | 956.67 | 426.99 | 1068.00 | 987.28 | 895.67 | 961.78 | 941.70 | 986.89 |
|  | **993.17** | **638.59** | **862.72** | **829.29** | **449.34** | **1020.50** | **895.42** | **946.61** | **949.17** | **961.10** | **848.19** |
|  | **52.47** | **34.02** | **30.65** | **64.34** | **46.38** | **43.89** | **189.92** | **82.39** | **17.27** | **69.15** | **177.35** |
|  | **6.00** | **6.00** | **6.00** | **6.00** | **6.00** | **6.00** | **6.00** | **6.00** | **6.00** | **6.00** | **6.00** |
|  | **21.42** | **13.89** | **12.51** | **26.27** | **18.93** | **17.92** | **77.53** | **33.64** | **7.05** | **28.23** | **72.40** |
|  |  |  |  |  |  |  |  |  |  |  |  |
| **SOD** | Intact control | Negative control | Silymarin 100 mg/kg + PCM | Pheophytin-A 100 mg/kg + PCM | Flavonol glycoside 100 mg/kg + PCM | Quercetin 100 mg/kg + PCM | Mother Liquor 400 mg/kg + PCM | n-Butanol Extract 400 mg/kg + PCM | Ethyl Acetate Extract 400 mg/kg + PCM | Chloroform Extract 400 mg/kg + PCM | n-Hexane Extract 400 mg/kg + PCM |
|  | 191.50 | 123.22 | 141.36 | 140.29 | 181.61 | 207.50 | 62.41 | 135.00 | 89.08 | 76.28 | 178.41 |
|  | 216.04 | 129.62 | 212.84 | 130.69 | 145.63 | 222.44 | 126.42 | 179.77 | 158.43 | 99.75 | 250.18 |
|  | 204.69 | 125.74 | 171.86 | 135.11 | 110.24 | 211.29 | 84.11 | 145.12 | 121.05 | 88.47 | 145.11 |
|  | 210.00 | 101.00 | 195.00 | 142.65 | 124.65 | 201.00 | 108.97 | 189.00 | 113.97 | 94.65 | 246.00 |
|  | 229.00 | 114.00 | 236.00 | 131.67 | 149.67 | 215.00 | 118.27 | 139.45 | 99.67 | 86.94 | 194.00 |
|  | 212.38 | 118.94 | 185.34 | 128.65 | 151.69 | 217.89 | 79.24 | 151.97 | 109.67 | 79.94 | 224.65 |
|  | **210.60** | **118.75** | **190.40** | **134.84** | **143.92** | **212.52** | **96.57** | **156.72** | **115.31** | **87.67** | **206.39** |
|  | **12.42** | **10.24** | **32.80** | **5.59** | **24.59** | **7.65** | **25.05** | **22.36** | **23.91** | **8.78** | **41.26** |
|  | **6.00** | **6.00** | **6.00** | **6.00** | **6.00** | **6.00** | **6.00** | **6.00** | **6.00** | **6.00** | **6.00** |
|  | **5.07** | **4.18** | **13.39** | **2.28** | **10.04** | **3.12** | **10.23** | **9.13** | **9.76** | **3.58** | **16.84** |
|  |  |  |  |  |  |  |  |  |  |  |  |
| **GR** | Intact control | Negative control | Silymarin 100 mg/kg + PCM | Pheophytin-A 100 mg/kg + PCM | Flavonol glycoside 100 mg/kg + PCM | Quercetin 100 mg/kg + PCM | Mother Liquor 400 mg/kg + PCM | n-Butanol Extract 400 mg/kg + PCM | Ethyl Acetate Extract 400 mg/kg + PCM | Chloroform Extract 400 mg/kg + PCM | n-Hexane Extract 400 mg/kg + PCM |
|  | 0.32 | 0.13 | 0.01 | 0.03 | 0.04 | 0.05 | 0.04 | 0.34 | 0.19 | 0.03 | 0.06 |
|  | 0.4 | 0.11 | 0.12 | 0.02 | 0.03 | 0.024 | 0.05 | 0.16 | 0.13 | 0.05 | 0.01 |
|  | 0.36 | 0.09 | 0.05 | 0.01 | 0.031 | 0.03 | 0.06 | 0.21 | 0.139 | 0.02 | 0.08 |
|  | 0.45 | 0.07 | 0.08 | 0.04 | 0.045 | 0.03 | 0.03 | 0.19 | 0.17 | 0.07 | 0.06 |
|  | 0.4 | 0.12 | 0.08 | 0.02 | 0.02 | 0.03 | 0.05 | 0.32 | 0.14 | 0.06 | 0.064 |
|  | 0.29 | 0.18 | 0.08 | 0.07 | 0.05 | 0.04 | 0.06 | 0.34 | 0.19 | 0.067 | 0.09 |
|  | **0.37** | **0.12** | **0.07** | **0.03** | **0.04** | **0.03** | **0.05** | **0.26** | **0.16** | **0.05** | **0.06** |
|  | **0.06** | **0.04** | **0.04** | **0.02** | **0.01** | **0.01** | **0.01** | **0.08** | **0.03** | **0.02** | **0.03** |
|  | **6.00** | **6.00** | **6.00** | **6.00** | **6.00** | **6.00** | **6.00** | **6.00** | **6.00** | **6.00** | **6.00** |
|  | **0.02** | **0.02** | **0.02** | **0.01** | **0.00** | **0.00** | **0.00** | **0.03** | **0.01** | **0.01** | **0.01** |
|  |  |  |  |  |  |  |  |  |  |  |  |
| **LP** | Intact control | Negative control | Silymarin 100 mg/kg + PCM | Pheophytin-A 100 mg/kg + PCM | Flavonol glycoside 100 mg/kg + PCM | Quercetin 100 mg/kg + PCM | Mother Liquor 400 mg/kg + PCM | n-Butanol Extract 400 mg/kg + PCM | Ethyl Acetate Extract 400 mg/kg + PCM | Chloroform Extract 400 mg/kg + PCM | n-Hexane Extract 400 mg/kg + PCM |
|  | 4.96 | 3.15 | 6.96 | 8 | 3.34 | 6.84 | 7.5 | 8.06 | 8.43 | 7.18 | 8.12 |
|  | 8.75 | 9.12 | 4.87 | 7.43 | 7.25 | 8.06 | 8 | 8.62 | 11.4 | 8.43 | 5.5 |
|  | 6.54 | 8.67 | 5.64 | 7.83 | 5.94 | 7.34 | 7.95 | 8.32 | 6.14 | 7.94 | 6.75 |
|  | 5.96 | 3.04 | 5.12 | 7.94 | 4.65 | 7.32 | 8.12 | 8.21 | 7.21 | 4.67 | 8.54 |
|  | 6.91 | 2.96 | 6.75 | 8.21 | 8.94 | 6.21 | 7.4 | 8.97 | 11.12 | 5.21 | 6.34 |
|  | 7.96 | 7.96 | 6.31 | 8.96 | 2.96 | 8.21 | 8.84 | 7.91 | 5.56 | 9.12 | 7.21 |
|  | **6.85** | **5.82** | **5.94** | **8.06** | **5.51** | **7.33** | **7.97** | **8.35** | **8.31** | **7.09** | **7.08** |
|  | **1.36** | **3.05** | **0.86** | **0.51** | **2.32** | **0.75** | **0.52** | **0.39** | **2.49** | **1.79** | **1.13** |
|  | **6.00** | **6.00** | **6.00** | **6.00** | **6.00** | **6.00** | **6.00** | **6.00** | **6.00** | **6.00** | **6.00** |
|  | **0.56** | **1.25** | **0.35** | **0.21** | **0.95** | **0.31** | **0.21** | **0.16** | **1.02** | **0.73** | **0.46** |
|  |  |  |  |  |  |  |  |  |  |  |  |
| **NO** | Intact control | Negative control | Silymarin 100 mg/kg + PCM | Pheophytin-A 100 mg/kg + PCM | Flavonol glycoside 100 mg/kg + PCM | Quercetin 100 mg/kg + PCM | Mother Liquor 400 mg/kg + PCM | n-Butanol Extract 400 mg/kg + PCM | Ethyl Acetate Extract 400 mg/kg + PCM | Chloroform Extract 400 mg/kg + PCM | n-Hexane Extract 400 mg/kg + PCM |
|  | 0.35 | 1.05 |  |  | 1.01 | 0.88 |  |  |  |  | 1.23 |
|  | 0.49 | 0.88 |  |  | 1.4 | 1.05 |  |  |  |  | 1.4 |
|  | 0.51 | 0.93 |  |  | 1.5 | 0.9 |  |  |  |  | 1.3 |
|  | 0.32 | 0.96 |  |  | 1.14 | 0.95 |  |  |  |  | 1.12 |
|  | 0.46 | 1.07 |  |  | 0.99 | 1.09 |  |  |  |  | 1.05 |
|  | 0.55 | 1.12 |  |  | 1.11 | 0.81 |  |  |  |  | 1.07 |
|  | **0.45** | **1.00** |  |  | **1.19** | **0.95** |  |  |  |  | **1.20** |
|  | **0.09** | **0.09** |  |  | **0.21** | **0.11** |  |  |  |  | **0.14** |
|  | **6.00** | **6.00** |  |  | **6.00** | **6.00** |  |  |  |  | **6.00** |
|  | **0.04** | **0.04** |  |  | **0.09** | **0.04** |  |  |  |  | **0.06** |

Table S4: Raw data of Table 4: Effects of *S. vermiculata* extracts and isolated compounds on inflammatory markers in PCM-induced liver toxicity in experimental mice

| **IL6** | Intact control | Negative control | Silymarin 100 mg/kg + PCM | Pheophytin-A 100 mg/kg + PCM | Flavonol glycoside 100 mg/kg + PCM | Quercetin 100 mg/kg + PCM | Mother Liquor 400 mg/kg + PCM | n-Butanol Extract 400 mg/kg + PCM | Ethyl Acetate Extract 400 mg/kg + PCM | Chloroform Extract 400 mg/kg + PCM | n-Hexane Extract 400 mg/kg + PCM |
| --- | --- | --- | --- | --- | --- | --- | --- | --- | --- | --- | --- |
|  | 6125.33 | 5818.67 | 5925.33 | 5805.33 | 5898.67 | 5792 | 6205.33 | 5885.33 | 5992 | 5925.33 | 6072 |
|  | 6072 | 5765.33 | 6032 | 5672 | 6272 | 5925.33 | 6178.67 | 5832 | 5872 | 5912 | 6072 |
|  | 6032 | 6232 | 6165.33 | 5738.67 | 5778.67 | 5898.67 | 6178.67 | 5952 | 5992 | 6432 | 5992 |
|  | 6272 | 5858.67 | 6058.67 | 5771.94 | 6194.84 | 6165.33 | 6171.65 | 5870.34 | 5914.34 | 5978.67 | 6012.67 |
|  | 6098.65 | 6134.54 | 6034.98 | 5994.64 | 5849.79 | 5894.64 | 6054.65 | 5891.94 | 5874.21 | 6234.32 | 5891.48 |
|  | 6105.78 | 5708.98 | 5974.64 | 6132.64 | 5976.12 | 6064.48 | 6214.67 | 5832.64 | 5776.64 | 5941.46 | 6054.74 |
|  | **6117.63** | **5919.70** | **6031.83** | **5852.54** | **5995.02** | **5956.74** | **6167.27** | **5877.38** | **5903.53** | **6070.63** | **6015.82** |
|  | **82.22** | **212.53** | **81.44** | **174.89** | **197.07** | **134.52** | **57.72** | **44.62** | **82.13** | **213.92** | **69.09** |
|  | **6.00** | **6.00** | **6.00** | **6.00** | **6.00** | **6.00** | **6.00** | **6.00** | **6.00** | **6.00** | **6.00** |
|  | **33.57** | **86.77** | **33.25** | **71.40** | **80.45** | **54.92** | **23.56** | **18.22** | **33.53** | **87.33** | **28.20** |
|  |  |  |  |  |  |  |  |  |  |  |  |
|  |  |  |  |  |  |  |  |  |  |  |  |
|  |  |  |  |  |  |  |  |  |  |  |  |
| **TNFa** | Intact control | Negative control | Silymarin 100 mg/kg + PCM | Pheophytin-A 100 mg/kg + PCM | Flavonol glycoside 100 mg/kg + PCM | Quercetin 100 mg/kg + PCM | Mother Liquor 400 mg/kg + PCM | n-Butanol Extract 400 mg/kg + PCM | Ethyl Acetate Extract 400 mg/kg + PCM | Chloroform Extract 400 mg/kg + PCM | n-Hexane Extract 400 mg/kg + PCM |
|  | 7512.24 | 7058.51 | 7386.87 | 7106.27 | 7392.84 | 7589.85 | 7297.31 | 7010.75 | 7022.69 | 7333.13 | 7482.39 |
|  | 7637.61 | 7212.39 | 7315.22 | 7106.27 | 7351.04 | 7243.58 | 7560 | 7118.21 | 7112.24 | 7434.63 | 7416.72 |
|  | 7579.4 | 7174.93 | 7607.76 | 7225.67 | 7291.34 | 7542.09 | 7488.36 | 7219.7 | 7028.66 | 7070.45 | 7189.85 |
|  | 7452.54 | 7133.13 | 7315.22 | 7210.14 | 7415.64 | 7136.12 | 7465.21 | 7025.65 | 7004.94 | 7667.46 | 7108.64 |
|  | 7689.34 | 7001.21 | 7065.34 | 7145.61 | 7124.64 | 7596.38 | 7021.24 | 7295.65 | 7105.67 | 7041.64 | 7349.54 |
|  | 7551.48 | 7068.64 | 7146.21 | 7121.64 | 7249.64 | 7089.64 | 7145.64 | 7158.64 | 7156.34 | 7516.94 | 7401.54 |
|  | **7570.44** | **7108.14** | **7306.10** | **7152.60** | **7304.19** | **7366.28** | **7329.63** | **7138.10** | **7071.76** | **7344.04** | **7324.78** |
|  | **85.30** | **79.26** | **190.38** | **52.82** | **107.57** | **235.96** | **212.91** | **110.60** | **61.11** | **248.64** | **144.72** |
|  | **6.00** | **6.00** | **6.00** | **6.00** | **6.00** | **6.00** | **6.00** | **6.00** | **6.00** | **6.00** | **6.00** |
|  | **34.82** | **32.36** | **77.72** | **21.56** | **43.91** | **96.33** | **86.92** | **45.15** | **24.95** | **101.51** | **59.08** |

Figure S1: Proton NMR of Compound 2, isorhamnetin-3-O-rutinoside

Figure S2: ^13^C-NMR of compound 2, isorhamnetin-3-O-rutinoside

Figure S3: MS spectra of compound 2 (isorhamnetin-3-O-rutinoside)

Figure S4: Proton NMR of compound 3, quercetin

Figure S5: ^13^C-NMR of compound 3, quercetin

Figure S6: MS spectra of tcompound 3 (quercetin)

Figure S7: Proton NMR of compound 1, Pheophytin A

Figure S8: ^13^C-NMR of compound 1, Pheophytin A


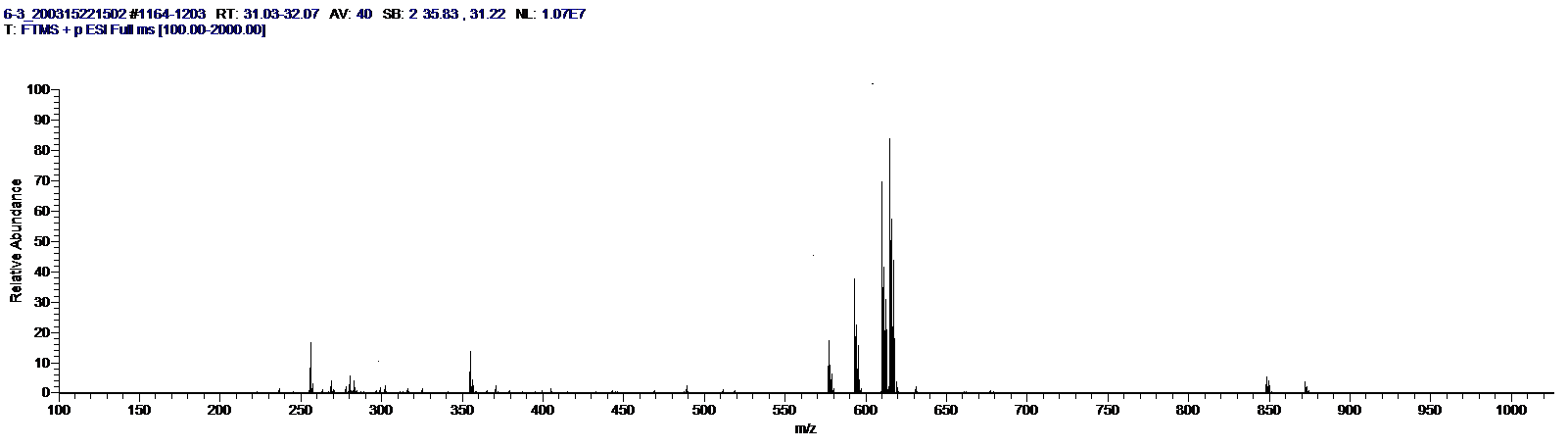


Figure S9: MS spectra of the compound 1, Pheophytin A

Scheme 1: Extraction, fractionation and isolation of the major compounds of *Suaeda vermiculata* herb
