# Supplementary figures and images for: Roles of Suaeda vermiculata Aqueous-Ethanolic Extract, Its Subsequent Fractions, and the Isolated Compounds in Hepatoprotection against Paracetamol-Induced Toxicity as Compared to Silymarin
Source: Oxid Med Cell Longev. 2021 Sep 17;2021:6174897. doi: 10.1155/2021/6174897 (PMC8463249; doi:10.1155/2021/6174897)

2-3 #760-765 RT: 20.24-20.38 AV: 6 SB: 2 23.02 , 18.96 NL: 5.60E4

T: FTMS - p ESI Full ms [100.00-2000.00]

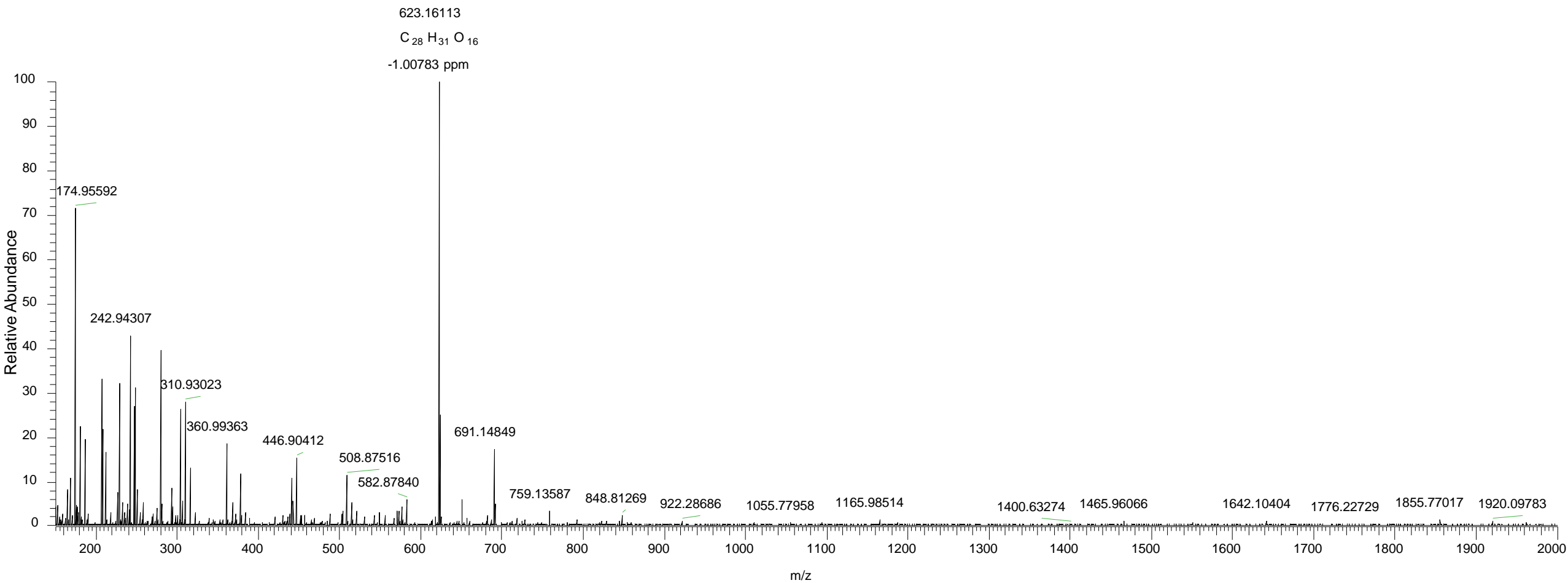

Supplement: Supplementary Materials — Supplementary file includes four tables (Tables S1 to S4) that describe the raw data related to the demonstrated biological activities of S. vermiculata. The file also includes nine figures (Figures S1 to S9) that showed the NMR and mass spectra of the isolated compounds. Besides, one scheme that describes the extraction and chromatographic separation of isolated compounds is also provided in the Supplementary file. [file 6174897.f1.zip › Figure S3.pdf]

1-3\_200315185024 #813-822 RT:21.66-21.90 AV: 10 SB: 2 22.54 , 21.26 NL: 9.20E5

F: FTMS + p ESI Full ms [100.00-2000.00]

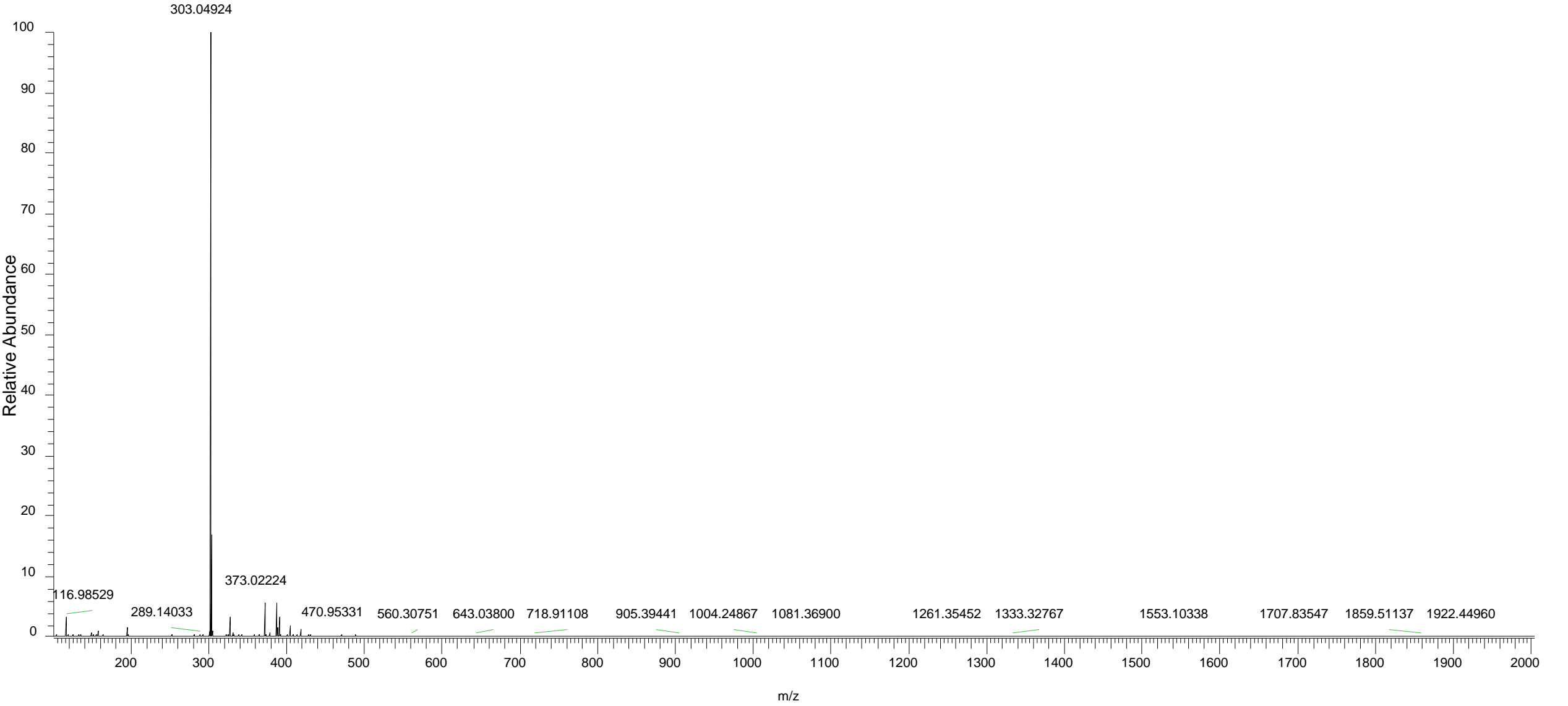

Supplement: Supplementary Materials — Supplementary file includes four tables (Tables S1 to S4) that describe the raw data related to the demonstrated biological activities of S. vermiculata. The file also includes nine figures (Figures S1 to S9) that showed the NMR and mass spectra of the isolated compounds. Besides, one scheme that describes the extraction and chromatographic separation of isolated compounds is also provided in the Supplementary file. [file 6174897.f1.zip › Figure S6.pdf]

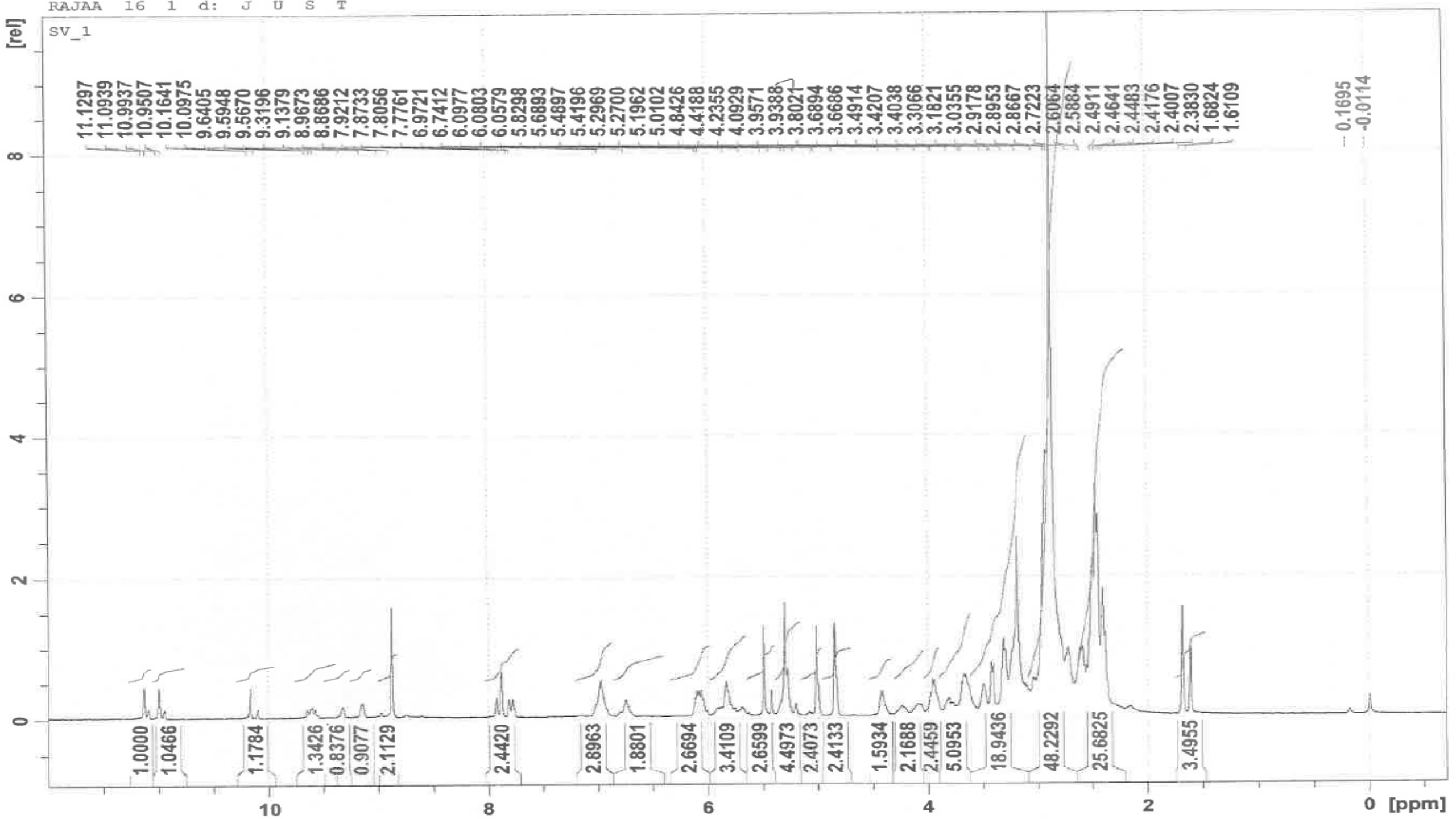

Supplement: Supplementary Materials — Supplementary file includes four tables (Tables S1 to S4) that describe the raw data related to the demonstrated biological activities of S. vermiculata. The file also includes nine figures (Figures S1 to S9) that showed the NMR and mass spectra of the isolated compounds. Besides, one scheme that describes the extraction and chromatographic separation of isolated compounds is also provided in the Supplementary file. [file 6174897.f1.zip › Figure S7.pdf]

6-3\_200315221502#1164-1203 RT: 31.03-32.07 AV: 40 SB: 2 35.83, 31.22 NL: 1.07E7

T: FTMS+pESI Full ms [100.00-2000.00]

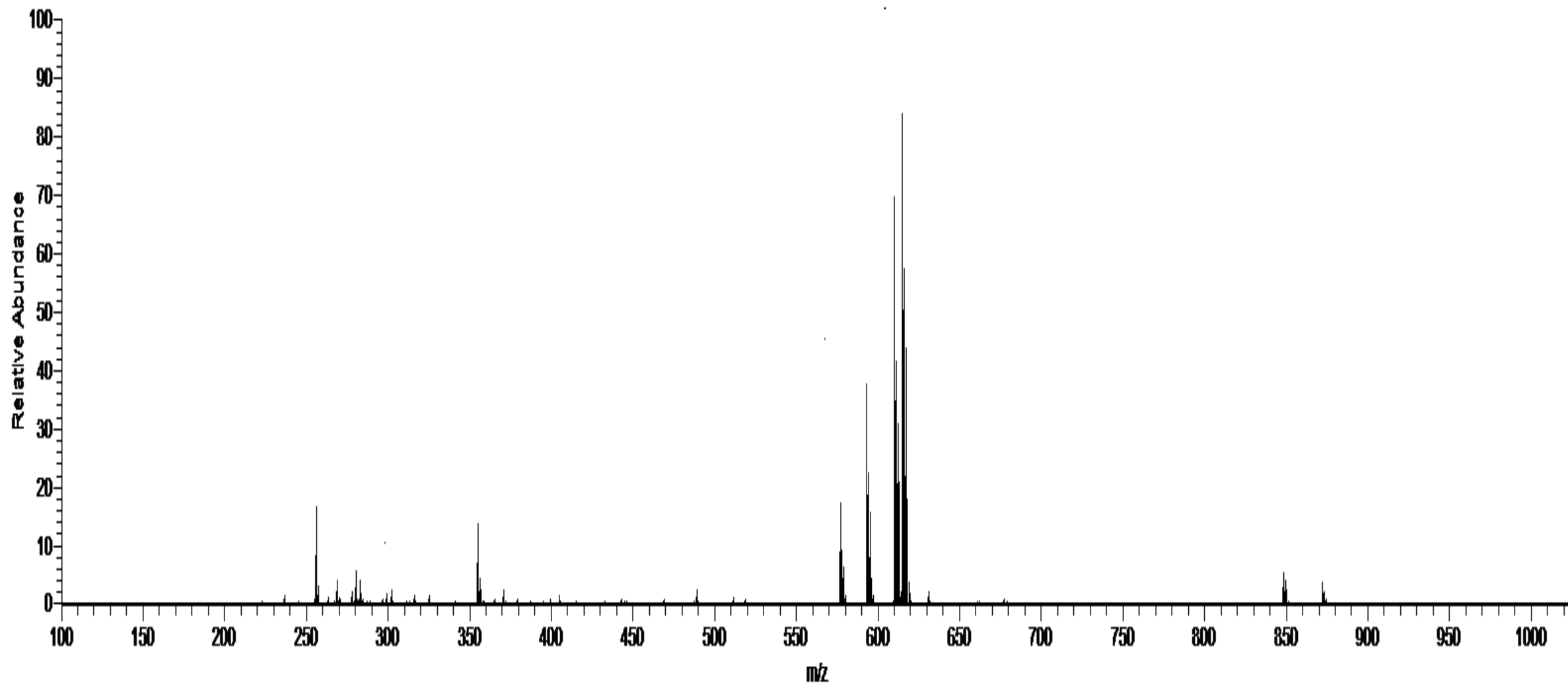

Supplement: Supplementary Materials — Supplementary file includes four tables (Tables S1 to S4) that describe the raw data related to the demonstrated biological activities of S. vermiculata. The file also includes nine figures (Figures S1 to S9) that showed the NMR and mass spectra of the isolated compounds. Besides, one scheme that describes the extraction and chromatographic separation of isolated compounds is also provided in the Supplementary file. [file 6174897.f1.zip › Figure S9.pdf]
